# Swimming championship finalist positions on success in international swimming competitions

**DOI:** 10.1371/journal.pone.0187462

**Published:** 2017-11-06

**Authors:** I. Yustres, R. Martín, L. Fernández, J. M. González-Ravé

**Affiliations:** 1 Department of Physical Activity and Sport Sciences, University of Castilla la Mancha, Avda Carlos III, Toledo, Spain; 2 Department of Mathematics, University of Castilla la Mancha, Avda Carlos III, Toledo, Spain; Nanyang Technological University, SINGAPORE

## Abstract

The primary goal was to determine whether the achievement of finalist positions in the Junior Championship was associated with the achievement of success in the International Swimming Federation (FINA) World Championship (WC). Secondary goals included analyzing the effect of various factors (gender, age, country, etc) on swimmers’ performances. Data were obtained from FINA information about the finalists from 2007 to 2015 WCs and finalists from 2006 to 2013 Junior-WCs (2400 entries). Final filtered database just included swimmers who participated in both junior and senior WCs (719 entries). A univariate general linear model (GLM) was used to examine the association between time; origin (swimmer who participated in Junior WC or not); maintenance years (number of years achieving finalist positions); country; and age, adjusting for year of competition. An ordinal logistic regression (OLR) model was used to identify predictors of achieving the top positions. The origin variable was not significant in either the GLM or the OLR. The only significant variables in the GLM were maintenance years (F_4,706_ = 7.689; p < .05) and year of competition (F_4,706_ = 23.239; p < .05). The OLR revealed a strong association (p < .001) between the position variable and maintenance years, getting better positions as you get more WCs (odds = 1.85). In conclusion, no evidence was obtained to conclude finalist position in Junior WC have influence in achieve success in FINA WC. Maintenance years in WCs have a positive impact to achieve better positions.

## Introduction

Many factors affect talent development in swimming categories, most of which are studied for performance optimization [1].

Although there is a rather long list of research studies about different aspects that affect the performance optimization, little is known, however, about the influence of performance in world-class junior categories on results in senior ones [2]. Trying to answer this question, different positions have been found in the development of elite athletes across a variety of sports [3], or analyzing the performance stability during elite swimmers’ careers in specific countries [4,5].

On the one hand, Allen et al. [5] showed that cumulative training hours are important for talent development at an early age in swimmers. In addition, some degree of sports specialization is necessary to develop elite-level skill development [6].

On the other hand, it has been found that an earlier onset and a higher volume of discipline-specific training and competition, and an extended involvement in institutional talent promotion programs, during adolescence need not necessarily be associated with greater success in senior international elite sport [7]. Also has been found a low conversion rate from elite junior athletes to elite senior athletes in swimming [8]. A third of international pre-junior athletes reappeared as senior athletes [9], confirming the difficulties of predicting later success based on early identification and selection.

Besides, it has been suggested that performance progression trajectories are generally non-linear, with a poorly predictable pattern, and with complex ascending and descending oscillations [3]. Costa et al. [4] also found that based on overall tracking values from childhood to adulthood, swimmers have a constantly changing performance trajectory.

The potential benefits of specialized sports at an early age in light of the potential risks associated with specialized participation focused on teenagers and criticized the gold medals and world records awarded to athletes in their early to mid-teenage years have been also discussed [10]. Adding Post et al. [11], showed that significant associations exist between sport specialization and injury history in a nonclinical population-based setting.

Costa et al. [12] tracked the performance trajectories of world-ranked swimmers affirming that coaches should have a long term view in what concerns training design and periodization. Also Allen et al. [13] assessed the career performance trajectories of Olympic swimmers to determine benchmarks for talent development showing that men achieved peak performance later than women (24.2 ± 2.1 vs. 22.5 ± 2.4 years).

As it was shown, there is a wide range of controversial outcomes obtained from diverse studies that have examined this question in one form or another, focusing each of them on some specific samples [4,12,14, 15]. But to date, there is still a lack of knowledge about the influence of performance in junior categories on results in senior categories focused on world elite population in swimming in a multivariate way (taking into account maintenance years, year of competition, age and country. A standardization process was carried out to remove the effect of the variables: distance, swim stroke, and gender on the swimmers’ marks).

This study´s landmark investigation draws from Junior and Senior FINA World Championships´ results from 2006 (first Junior World Championship) to 2015. Being the main goal of this research, to determine whether the achievement of finalist positions in the Junior Championship was associated with the achievement of success in the FINA World Championships between 2007 and 2015. Therefore, we hypothesized that the achievement of an optimal performance in Junior World Championships positively affects the achievement of performance in World Championships in senior category.

## Methods

To assess the relationship between the achievement of finalist position in Junior World Championships and the achievement of success in the final of Senior World Championships, an observational retrospective study was conducted. Thus, we used historical data retrieving the information from official databases between 2007 and 2015.

### Data collection procedure

Data (2400 entries) for the present study were derived from the International Junior Swimming Categories (http://www.fina.org/results). Raw data (2400) was divided first in two differentiated databases. Finalists in the World Championships in 2007, 2009, 2011, 2013 and 2015 (database 1) and finalist in the Junior World Championships in 2006, 2008, 2011 and 2013 (database 2). Each entry contains the following information: Full name, ID, time (mark), position (1st to 8th), birth year, country, sex, distance, swim stroke, maintenance years and year of competition. Being the distances analyzed 50, 100, 200, 400, 800, 1500 freestyle; 50, 100, 200 backstroke/breaststroke/butterfly and 200, 400 individual medley.

The Castilla-La Mancha University Ethical Committee approved this research dated November 30^th^ 2016. Informed consent from participants were not necessary because we used public data uploaded on internet.

### Sampling and study design

For our purpose, database 2 was filter and sort, selecting only swimmers who repeat the occurrence in database 1 getting then a total of 1360 entries from both databases.

The dichotomous variable, origin, was added to database 1 by comparing the two databases and assigning 0 (No Junior: finalist positions in World Championships) when the subject appeared only in database 1, or 1 (Junior: finalist positions in Junior and World Championships) when the subject appeared in both databases.

On the other hand, the maintenance years factor shows the number of years that the swimmer has remained in the elite participating in world championships until the year of the world competition in which participates.

Taking into account that the first Junior World Championships took place in 2006, the data were restricted according to swimmers’ age to include only those who could have participated in the Junior category due to their year of birth. This was necessary to prevent assigning a No Junior category to a swimmer whose age did not allow him to participate in a Junior championship. This restriction prevented incorrectly counting the maintenance years of a swimmer who was finalist in some World Championships before 2007. Therefore, 641 entries were removed for the swimmers who fulfilled one of the following conditions: a) Male: birth year <1987; b) Female: birth year <1989. Thus, a total 719 entries were included in the final filtered database.

Two response variables were employed to measure the performance: the continuous variable, time, measured in hundredths of a second; and the ordinal variable, position (Table 1).

**Table 1:** Independent variables and their levels.

Variable	Levels
Origin	Junior, No Junior
Maintenance Years	1, 2, 3, ≥4 (years)
Swim stroke	Styles, Freestyle, Backstroke, Breaststroke, Butterfly
Distance	50, 100, 200, 400, 800, 1500 (metres)
Gender	Male, Female
Age	15–16, 17–18, 19–20, 21–22, 23–24, 25–26, 27–28
Country	40 different countries
Year Competition	2007, 2009, 2011, 2013, 2015

### Statistical analysis

Descriptive statistics were employed for the study to examine the explicative variables. Double entry boxes were used to analyze the distribution of the date comparing origin with gender, maintenance years and position. The information between distances, origin and gender were compared using a triple-entry table.

A univariate general linear model (GLM) was used to examine the association between time, origin, maintenance years, country and age, adjusting for year of competition. The last is a blocking variable included as a possible nuisance factor whose effect must be controlled. To compare the swimmers’ marks, taking into account the distances, swim strokes and sex, the time variable was standardized in each group (there were 60 = 6·5·2 total groups). These variables were not included in the model because the number of factors and correlations would be too high, thus not enough data would be in each group. Therefore, the variable ZTime was considered to be the dependent variable in the model. Model assumptions of normality, homoscedasticity and independence were tested using the Kolmogorov-Smirnov test, Levene’s test for homoscedasticity and the test for independence, respectively. All the residuals showed a satisfactory pattern. Pairwise *post hoc* comparisons using Bonferroni adjustments were performed using estimated marginal means. Effect-size analyses for univariate general lineal model (GLM) were calculated using partial eta-squared, η^2^_p_, while for t-tests were calculated using Cohen’s effect [16,17]. By convention, effect sizes < .01, .01-.09, .09-.25, ≥0.25 were considered negligible, small, moderate and large effects for η^2^_p_, and < .2, .2-.5, .5-.8, ≥.8 for Cohen’s d effect respectively [16]. To measure performance by position, an ordinal logistic regression model (OLR) was used to find predictors of achieving the top positions. The ordered logistic model is an extension of the binary response model. A wide variety of ordered or ordinal response models have been developed. The foremost ordinal logistic model used in practice is the proportional odds model. This model assumes that the model coefficients for each level or response are equal. The test of parallel lines was employed to see whether the primary model assumption was violated. The data satisfied the proportional odds assumption; therefore, this model was used in this study. Statistical significance was set at p < .05. Statistical analysis was carried out using SPSS statistical software (version 21, IBM).

## Results

Of 719 entries included in our study, only 17% were finalists in both championships (Junior category); in contrast, 83% of the entries were included in the No Junior category. A total of 55.3% and 50% were female and 44.7% and 50% male (Junior and No Junior respectively), with a mean age of 20.04 ± 2.21 years for Junior and 21.47 ± 2.63 years for No Junior, ranging from 15 to 25 years the swimmers from Junior and from 15 to 28 years those from No Junior. Mean maintenance in the elite was of 1.85 ± 0.85 and 2.42 ± 1.28. (Table 2).

**Table 2:** Characterization of the sample (n = 719).

**Variable**	** **	**Junior**	**No Junior**
***N***		123 (17.1%)	596 (82.9%)
***Gender***	**Male**	55 (44.7%)	298 (50%)
	**Female**	68 (55.3%)	298 (50%)
***Year Competition***	**2007**	3 (2.4%)	29 (4.9%)
	**2009**	14 (11.4%)	84 (14.1%)
	**2011**	21 (17.1%)	126 (21.1%)
	**2013**	45 (36.6%)	163 (27.3%)
	**2015**	40 (32.5%)	194 (32.6%)
***Age [years]***;***Mean ± SD Range***		20.04 ±2.21(15 to 25)	21.47 ±2.63(15 to 28)

***Maintenance Years***;***Mean ± SD Range***		1.85 ±0.85(1 to 4)	2.42 ±1.28(1 to 5)
**Variable**	** **	**Junior**	**No Junior**
***N***		123 (17.1%)	596 (82.9%)
***Gender***	**Male**	55 (44.7%)	298 (50%)
	**Female**	68 (55.3%)	298 (50%)
***Year Competition***	**2007**	3 (2.4%)	29 (4.9%)
	**2009**	14 (11.4%)	84 (14.1%)
	**2011**	21 (17.1%)	126 (21.1%)
	**2013**	45 (36.6%)	163 (27.3%)
	**2015**	40 (32.5%)	194 (32.6%)
***Age [years]***;***Mean ± SD Range***		20.04 ±2.21(15 to 25)	21.47 ±2.63(15 to 28)

***Maintenance Years***;***Mean ± SD Range***		1.85 ±0.85(1 to 4)	2.42 ±1.28(1 to 5)

Firstly, focusing on the variable origin and its interaction with some of the variables, the percentage data for men who came from the No Junior category was 84.4% and 81.4% for women. The Junior category showed a percentage of men of 44.7%, compared with 50% in the No Junior category.

Regarding the maintenance years variable, a large percentage of marks were related to No Junior swimmers (80.2%), who had been in the world elite only for 1 year. This finding is reasonable because of the large sample size of the No Junior group. However, only 52.2% of No Junior entries had been in the elite for one year versus 62.6% for those who came from Junior category. In the second maintenance year, the results were more similar between groups: 28.5% Junior compared with 27.5% No Junior. In the third and fourth maintenance years, the Junior percentage decreased considerably (8.1% and 0.8%, respectively) compared with the No Junior category (12.8% and 6%).

The number of data from the Junior category increases every year of competition; however, a far larger proportion of participants from the No Junior group was observed; for example, in 2007, the ratio was 9.4% Junior to 90.6% No Junior.

Concerning the distances, the largest proportion of data was observed in the 200 m., whereas the lowest was in the 1500 m. In particular, marks from the Junior category (85.7%) men’s backstroke in the 200 m. distance are remarkable.

On the other hand, several models were created in order to analyze the performance with Ztime (GLM) and position (OLR) as dependent variables.

### Performance by ZTime (GLM)

**Fig 1:**
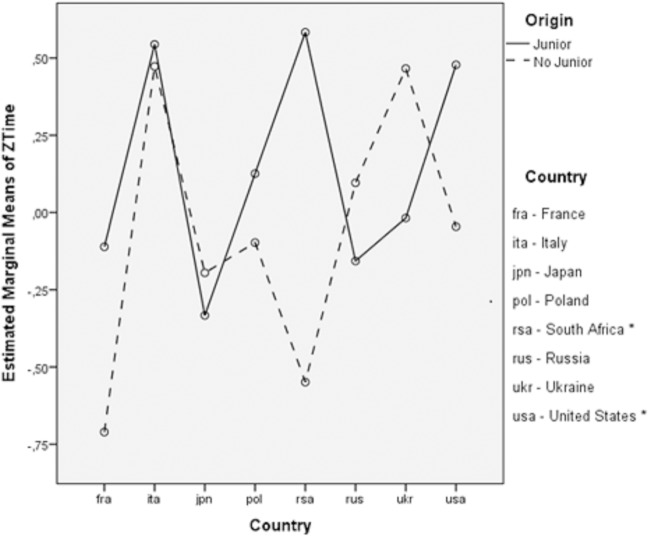
Profile plots for estimated marginal means of *ZTime*: Origin*Country. (*Significant differences at p < 0.05 between Junior and No Junior groups).

The only significant primary effect in this model was maintenance years (F_4,706_ = 7.689; *p* < .001, η^2^_p_ = .04) although the effect size was small; however, the year of competition was a significant block variable (F_4,706_ = 23.239; *p* < .001, η_p_ = .12) with a moderate effect size. The remaining primary effects (origin *p* = .93 - η_p_ < .01, age *p* = .80 - η_p_ = .01, country *p* = .18 -—η_p_ = .07) were not statistically significant (*p >* .05), and moderate effect size was observed for country. Although the origin variable was not statistically significant, for the purpose of this study, this variable was included in the model in an attempt to determine correlations with other factors. It is worth emphasizing that a weak statistical interaction between origin and country was found. The results of the analysis of variance showed an F_4,706_ = 1.15; p = .07; η_p_ = .04. Therefore, the analysis was repeated, including only a selection of countries that met the criterion of accounting for at least 20% of the overall data. This criterion was necessary due to the low participation of swimmers from the Junior category. As an exception, the United States was included in this analysis, despite having a proportion of 14% in this category (<20%), due to the large number of inputs (16) compared with the rest of the countries. Once the significance of the interaction between origin and country was again verified in the model with the selection of countries, multiple comparisons by the Bonferroni method showed that the only significant difference between Junior and No Junior groups were in South Africa (p = .02, d = 1.51) and the United States (p = .03, d = .49). The mean performance time of the Junior group in these countries was significantly higher than the mean of No Junior (Fig 1).

Focusing now on position as response variable to measure the performance, our study shows:

### Performance by position (ORL)

Before building the ordinal logistic regression model, the data were examined through double entry tables and cumulative percentage graphs to identify possible differences between factor levels, which could explain position variability.

#### Origin and position

**Fig 2:**
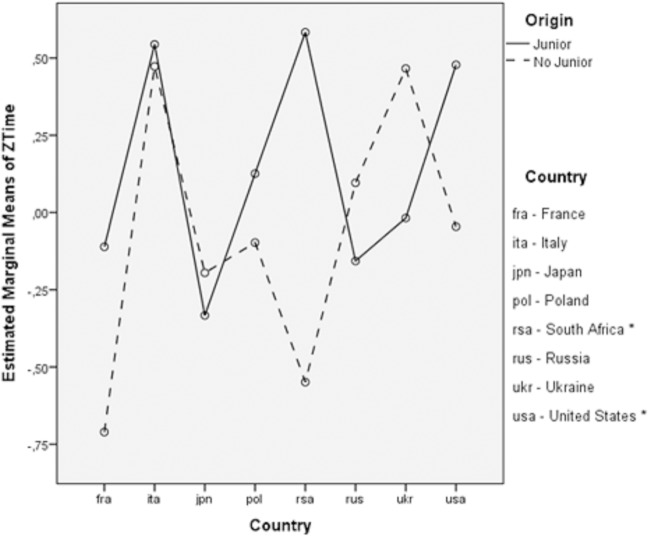
Cumulative percentage plots for *Position* in separate lines for *Origin* levels.

The proportion of No Junior data, which reached top positions, was higher (36%) than that of the Junior category (32.5%). Furthermore, the results of the last 3 positions showed a higher percentage in the Junior category (47.9% Junior compared with 37.1% No Junior). Swimmers from Junior category primarily held the last positions (Fig 2).

#### Maintenance years and position

**Fig 3:**
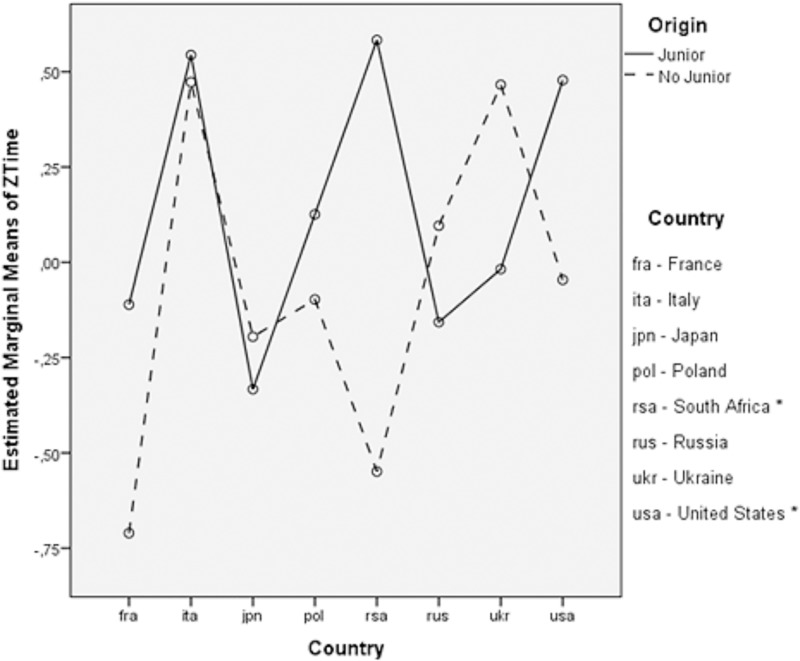
Cumulative percentage plots for *Position* in separate lines for *Maintenance Years* levels.

There exists a clear relationship between maintenance years in the world elite and the position. The cumulative percentage in the last positions is higher for data relating to a lower number of maintenance years; this percentage is reduced when the number of maintenance years is higher. Thus, approximately 50% of 1 maintenance year’s data is accumulated in the three last positions, whereas approximately 70% of 4 or more maintenance years’ data is accumulated in the three top positions (Fig 3).

The same descriptive studies were carried out for all the other factors (sex, swim stroke, distance and age) failing to find any statistically significant evidence of effects. Thus, an ordinal logistic regression model was performed considering position as a dependent variable; and origin, sex, distance, swim stroke, country and maintenance year (which was introduced as a co variable) as independent variables. Sex, distance, swim stroke and country were not significant. Therefore, these variables and their possible interactions were extracted to the stepwise model (Table 3).

**Table 3:** Results of the proportional odds model using position as response eight ordered categories.

**Variables**	**Logistic coefficient**	**Standard error**	**p-value**	**95% CI**
**Position**				
**1**	-4.11	.27	p < 0.0001	-4.65, -3.56
**2**	-3.16	.26	p < 0.0001	-3.68, -2.65
**3**	-2.52	.25	p < 0.0001	-3.03, -2.02
**4**	-2.00	.25	p < 0.0001	-2.49, -1.50
**5**	-1.40	.24	p < 0.0001	-1.88, -.91
**6**	-.753	.24	p < 0.0001	-1.22, -.27
**7**	.070	.24	p = 0.77	-.40, .54
**Maintenance years**	-.612	.07	p < 0.0001	-0,75, -0.46
**Origin**				
*Junior*	-.18	.17	p = 0.28	-0.52, 0.15
*No Junior*	0^a^	-	-	-
**Link function: Logit.** a. The parameter is set to zero because it is redundant.				
**Variables**	**Logistic coefficient**	**Standard error**	**p-value**	**95% CI**
**Position**				
**1**	-4.11	.27	p < 0.0001	-4.65, -3.56
**2**	-3.16	.26	p < 0.0001	-3.68, -2.65
**3**	-2.52	.25	p < 0.0001	-3.03, -2.02
**4**	-2.00	.25	p < 0.0001	-2.49, -1.50
**5**	-1.40	.24	p < 0.0001	-1.88, -.91
**6**	-.753	.24	p < 0.0001	-1.22, -.27
**7**	.070	.24	p = 0.77	-.40, .54
**Maintenance years**	-.612	.07	p < 0.0001	-0,75, -0.46
**Origin**				
*Junior*	-.18	.17	p = 0.28	-0.52, 0.15
*No Junior*	0^a^	-	-	-
**Link function: Logit.** a. The parameter is set to zero because it is redundant.				

The model confirms there is a strong association between the position obtained and the years maintained in elite World Championships, getting better positions as you get more WCs. The odds of being entered to a higher position increase by 1/exp(-0.612) = 1.85 for every unit change in the maintenance years score.

## Discussion

The primary purpose of this research was to evaluate whether the achievement of finalist positions in the ranking of Junior Championships led to success in FINA World Championships from 2007 to 2015. We have compared the performance time results in FINA World Championships between the swimmers who competed priori in Junior squads and the swimmers who went straight to the Senior squads, showing no significant differences (p > .05) between them.

Bearing in mind our results, we do not find evidence to suggest that swimmers’ background does become a significant factor to get better results in senior categories, rejecting the hypothesis that the achievement of an optimal performance in Junior World Championships positively affects the achievement of performance in World Championships in senior category.

These results are aligned with some studies as Allen et al. [13] who affirm that as most swimmers selected to a national junior squad did not progress consistently through their developmental years to become national squad members and was also shown that the current trend for early specialization in junior international competitions might not be advantageous in the development of sports talent in swimming.

Also Gulbin et al. [3] found as trajectories of development are mostly characterized by non-linear patterns, with highly variable oscillations between and within the junior and senior competition levels. Adding, Costa et al. [4] also found evidence to suggest that the performance of sub-elite male freestyle swimmers does not become sufficiently stable to yield meaningful predictions of adult performance until age 16. Bringing account there is not a widespread opinion on this topic.

On the other hand, early success has been affirmed not to be the cause of later failures and drop-outs [18]. There are many more influential factors that affect motivation and the desire to participate that restrict the longevity of successful young swimmers in the sport.

Therefore, it would be interesting to explore the benefits and impacts of early participation of swimmers in international competitions in order to produce significant gains in swimming performance.

### Early specialization in world championships

According to Allen et al. [13] we did not find significant differences between maintenance years in senior FINA World Championships and origin. Moreover, our results found a strong association (p < .001) between the position variable and maintenance years, getting better positions as you get more World Championships (odds 1/exp(-0.612) = 1.85).

However, it is possible to attain an international senior level with less than 5 years of practice in the main sport and with a more diverse sport experience during early stages of development [19]. In contrast, the study of Barreiros et al. [9] investigated the international pathway of male and female athletes in various sports from the time of their competitive debut in order to determine how many international athletes competed or did not compete internationally at early ages as juniors and/or seniors. They found that only a third of international pre-junior athletes reappeared as senior athletes; being these results even but numerous than ours which has shown that only 17.1% of the swimmers have participated in Junior before to senior finalist World Championships.

The scarcity of participants coming from the Junior World Championship would suggest a methodological interference due to an unbalanced sample coming from junior and senior squads. Moreover, a survey of the tournaments is necessary to verify whether the results obtained in the present study confirm this tendency.

Focusing on the impacts that early specialization could have on swimmers, early emphasis on obtaining good results is associated with major rates of depletion and dropout when the athletes might be exposed to higher levels of pressure and stress [20]. Supporting therefore studies that the later the selection to a national team, the greater the development and sports performance in future categories [21, 22]. Being opposite to some authors who affirmed that swimming is considered an early specialization sport, in which extensive training must be performed from an early age in order to achieve long-term high performance [23]. Therefore, there is a rather long list of research studies having different perspectives but none of them provides the specific information for the total population of final swimmers in World Championships as in the current study, that´s the reason why carrying out individual case study approach for each country in which provide detailed information of the models of sports performance development used, could be interesting for incoming investigations.

A method to monitor performance progression of swim squads was demonstrated and used to assess the progression of New Zealand’s centralized elite swim squad [5]. Also four methods have been proposed [15] that retrospectively simulated early selection of swimmers into a talent development squad.

### Country and athlete’s development

On the other hand, the results showed that there are differences between the times depending on the year of competition. These differences might be due to regulation modification, such as for example the prohibition of polyurethane swimsuits from January 1, 2010. The prohibition came about because these swimsuits were leading to major buoyancy and minor resistance the water, which was translated in a notable way into better times achieved by the swimmers in the Senior FINA World Championship of 2009.

Also worth noting is the way the times obtained by the swimmers who did not take part in the Junior World Championships in the USA and the South African Republic were significantly lower compared with those swimmers who did take part. In this respect, it would be interesting to analyze whether both countries are presented with similar models of sports performance development that would help explain this statistical relationship as it has been showed in the study focused on the evaluation of the performance and progression of swimming teams in New Zealand [5].

Given the low coefficients of determination or pseudo-R2 found in all the models obtained in our study, other factors (not contemplated in our study) might explain the variability of time and position. The determination of these unassessed factors might be considered for a future study. The accomplishment of a longitudinal study with only the swimming participants in the Junior World Championship and assessing their progression in World Championship categories might also be considered for future studies.

## Conclusions

We conclude that finalist positions in Junior Championships have no influence in achieve success in FINA World Championships.

Maintenance years in World Championships have a positive impact to achieve better positions. Then, coaches might reconsider early specialization during the process as a means to achieve success in the highest levels of competition. On the other hand, swimming programs need to invest in strategies that keep swimmers in the sport for longer at the senior level as experience counts.

## Supporting information

S1 File Minimal data sheet. (PDF)
