# Supplementary material for: Swimming championship finalist positions on success in international swimming competitions
Source: PLoS One. 2017 Nov 6;12(11):e0187462. doi: 10.1371/journal.pone.0187462 (PMC5673220; doi:10.1371/journal.pone.0187462)
Supplement: S1 File — (PDF) [file pone.0187462.s001.pdf]

ID	Birthday	Country	Gender	Distance	Swim stroke	T1J2006
1	1989	ger	2	1	1	
2	1989	rom	2	1	1	
3	1989	fra	2	1	1	
4	1990	ukr	2	1	1	
5	1993	chn	2	1	1	
6	1991	swe	2	1	1	
7	1989	bra	2	1	1	
8	1989	fra	2	1	1	
9	1989	ger	2	2	1	
10	1989	rom	2	2	1	
11	1989	fra	2	2	1	
12	1993	chn	2	2	1	
13	1989	swe	2	2	1	
14	1990	ukr	2	2	1	
15	1989	gre	2	2	1	
16	1991	swe	2	2	1	
17	1990	fra	2	3	1	2
18	1993	chn	2	3	1	2
19	1990	usa	2	3	1	2
20	1989	bra	2	3	1	2
21	1990	usa	2	3	1	2
22	1990	chn	2	3	1	2
23	1989	ger	2	3	1	2
24	1990	esp	2	3	1	2
25	1990	esp	2	4	1	4
26	1990	usa	2	4	1	4
27	1990	usa	2	4	1	4
28	1990	rsa	2	4	1	4
29	1990	pol	2	4	1	4
30	1990	fra	2	4	1	4
31	1990	ita	2	4	1	4
32	1990	chn	2	4	1	4
33	1989	rom	2	5	1	8
34	1990	rsa	2	5	1	8
35	1989	rus	2	5	1	8
36	1990	fra	2	5	1	8
37	1990	slo	2	5	1	8
38	1991	gbr	2	5	1	8
39	1990	chn	2	5	1	8
40	1989	fra	2	5	1	8
41	1990	fra	2	6	1	16
42	1989	rus	2	6	1	16
43	1990	rsa	2	6	1	16
44	1990	usa	2	6	1	16
45	1989	fra	2	6	1	16
46	1990	slo	2	6	1	16
47	1990	chn	2	6	1	17
48	1990	mex	2	6	1	17
49	1990	chn	2	1	2	
50	1990	nzl	2	1	2	

51	1991 ger	2	1	2	
52	1990 rus	2	1	2	
53	1990 gbr	2	1	2	
54	1989 rsa	2	1	2	
55	1990 fra	2	1	2	
56	1991 chn	2	1	2	
57	1990 nzl	2	2	2	1
58	1990 rus	2	2	2	1
59	1990 chn	2	2	2	1
60	1990 fra	2	2	2	1
61	1989 rsa	2	2	2	1
62	1990 gbr	2	2	2	1
63	1992 pol	2	2	2	1
64	1990 rus	2	2	2	1
65	1990 rus	2	3	2	2
66	1990 fra	2	3	2	2
67	1991 chn	2	3	2	2
68	1990 rus	2	3	2	2
69	1990 usa	2	3	2	2
70	1989 rsa	2	3	2	2
71	1992 pol	2	3	2	2
72	1990 gbr	2	3	2	2
73	1990 chn	2	1	3	
74	1992 rus	2	1	3	
75	1990 chn	2	1	3	
76	1990 rus	2	1	3	
77	1989 ita	2	1	3	
78	1989 por	2	1	3	
79	1989 fra	2	1	3	
80	1990 pol	2	1	3	
81	1990 chn	2	2	3	1
82	1992 rus	2	2	3	1
83	1991 usa	2	2	3	1
84	1989 por	2	2	3	1
85	1990 rus	2	2	3	1
86	1989 swe	2	2	3	1
87	1990 pol	2	2	3	1
88	1989 fra	2	2	3	1
89	1992 rus	2	3	3	2
90	1990 chn	2	3	3	2
91	1991 usa	2	3	3	2
92	1990 rus	2	3	3	2
93	1989 rsa	2	3	3	2
94	1989 swe	2	3	3	2
95	1990 rsa	2	3	3	2
96	1990 pol	2	3	3	2
97	1989 ukr	2	1	4	
98	1990 ita	2	1	4	
99	1990 ger	2	1	4	
100	1989 aut	2	1	4	
101	1989 bra	2	1	4	

102	1990	chn	2	1	4	
103	1989	swe	2	1	4	
104	1990	gbr	2	1	4	
105	1990	ita	2	2	4	
106	1989	rsa	2	2	4	1
107	1990	gbr	2	2	4	1
108	1990	chn	2	2	4	1
109	1989	ukr	2	2	4	1
110	1989	svk	2	2	4	1
111	1990	fra	2	2	4	1
112	1990	ita	2	2	4	1
113	1990	usa	2	3	4	2
114	1990	gbr	2	3	4	2
115	1990	aut	2	3	4	2
116	1989	rsa	2	3	4	2
117	1990	chn	2	3	4	2
118	1989	svk	2	3	4	2
119	1989	ger	2	3	4	2
120	1990	usa	2	3	4	2
121	1991	usa	2	3	5	2
122	1989	fra	2	3	5	2
123	1990	chn	2	3	5	2
124	1990	rus	2	3	5	2
125	1990	rus	2	3	5	2
126	1989	rsa	2	3	5	2
127	1990	nzl	2	3	5	2
128	1990	swe	2	3	5	2
129	1990	esp	2	4	5	4
130	1989	rus	2	4	5	4
131	1989	rsa	2	4	5	4
132	1990	usa	2	4	5	4
133	1990	usa	2	4	5	4
134	1989	gre	2	4	5	4
135	1991	gbr	2	4	5	4
136	1990	pol	2	4	5	4
137	1989	bel	1	1	1	
138	1989	rus	1	1	1	
139	1989	rsa	1	1	1	
140	1990	nzl	1	1	1	
141	1989	ltu	1	1	1	
142	1989	ita	1	1	1	
143	1989	chn	1	1	1	
144	1990	ven	1	1	1	
145	1989	bel	1	2	1	
146	1989	rus	1	2	1	
147	1989	ita	1	2	1	
148	1990	ven	1	2	1	
149	1989	ukr	1	2	1	
150	1989	rsa	1	2	1	
151	1989	rsa	1	2	1	
152	1989	chn	1	2	1	

153	1989	ita	1	3	1	1
154	1989	bel	1	3	1	1
155	1989	usa	1	3	1	1
156	1989	mas	1	3	1	1
157	1989	bra	1	3	1	1
158	1990	gbr	1	3	1	1
159	1989	rus	1	3	1	1
160	1990	ita	1	3	1	1
161	1989	pol	1	4	1	3
162	1989	ita	1	4	1	3
163	1989	esp	1	4	1	3
164	1989	usa	1	4	1	3
165	1989	pol	1	4	1	3
166	1989	rus	1	4	1	3
167	1989	rsa	1	4	1	3
168	1989	chn	1	4	1	3
169	1989	pol	1	5	1	8
170	1989	esp	1	5	1	8
171	1989	ita	1	5	1	8
172	1989	usa	1	5	1	8
173	1989	rus	1	5	1	8
174	1989	ita	1	5	1	8
175	1989	rsa	1	5	1	8
176	1990	nzl	1	5	1	8
177	1989	pol	1	6	1	15
178	1989	esp	1	6	1	15
179	1989	usa	1	6	1	15
180	1989	ita	1	6	1	15
181	1989	usa	1	6	1	15
182	1990	nzl	1	6	1	15
183	1989	bra	1	6	1	15
184	1989	arg	1	6	1	15
185	1989	bra	1	1	2	
186	1989	ita	1	1	2	
187	1989	rsa	1	1	2	
188	1990	blr	1	1	2	
189	1990	rus	1	1	2	
190	1989	usa	1	1	2	
191	1989	cro	1	1	2	
192	1990	pol	1	1	2	
193	1989	ita	1	2	2	
194	1989	bra	1	2	2	
195	1990	usa	1	2	2	
196	1989	usa	1	2	2	
197	1990	rus	1	2	2	
198	1989	rsa	1	2	2	
199	1990	nzl	1	2	2	
200	1990	pol	1	2	2	
201	1989	usa	1	3	2	2
202	1989	ita	1	3	2	2
203	1989	usa	1	3	2	2

204	1990	gbr	1	3	2	2
205	1990	rus	1	3	2	2
206	1990	nzl	1	3	2	2
207	1989	gre	1	3	2	2
208	1989	pol	1	3	2	2
209	1989	ita	1	1	3	
210	1990	scg	1	1	3	
211	1989	pan	1	1	3	
212	1989	ita	1	1	3	
213	1989	slo	1	1	3	
214	1989	ukr	1	1	3	
215	1989	par	1	1	3	
216	1989	pol	1	1	3	
217	1989	ita	1	2	3	1
218	1989	ita	1	2	3	1
219	1989	rus	1	2	3	1
220	1990	rus	1	2	3	1
221	1989	ltu	1	2	3	1
222	1990	scg	1	2	3	1
223	1989	ukr	1	2	3	1
224	1989	kuw	1	2	3	1
225	1989	ita	1	3	3	2
226	1989	ita	1	3	3	2
227	1989	ltu	1	3	3	2
228	1990	rus	1	3	3	2
229	1989	bra	1	3	3	2
230	1989	kuw	1	3	3	2
231	1989	rus	1	3	3	2
232	1989	bra	1	3	3	2
233	1989	blr	1	1	4	
234	1989	bra	1	1	4	
235	1991	rus	1	1	4	
236	1989	mas	1	1	4	
237	1990	scg	1	1	4	
238	1989	bra	1	1	4	
239	1989	cro	1	1	4	
240	1989	rsa	1	1	4	
241	1990	scg	1	2	4	
242	1989	mas	1	2	4	
243	1991	rus	1	2	4	
244	1989	aut	1	2	4	
245	1989	blr	1	2	4	
246	1989	ukr	1	2	4	
247	1989	can	1	2	4	
248	1989	bra	1	2	4	
249	1989	aut	1	3	4	2
250	1989	mas	1	3	4	2
251	1989	ecu	1	3	4	2
252	1989	can	1	3	4	2
253	1990	per	1	3	4	2
254	1989	arg	1	3	4	2

255	1990	bra	1	3	4	2
256	1990	mex	1	3	4	2
257	1989	usa	1	3	5	2
258	1990	gbr	1	3	5	2
259	1989	ukr	1	3	5	2
260	1989	aut	1	3	5	2
261	1989	pol	1	3	5	2
262	1989	esp	1	3	5	2
263	1989	rus	1	3	5	2
264	1989	rsa	1	3	5	2
265	1989	usa	1	4	5	4
266	1989	pol	1	4	5	4
267	1989	aut	1	4	5	4
268	1990	gbr	1	4	5	4
269	1989	rsa	1	4	5	4
270	1989	esp	1	4	5	4
271	1989	rus	1	4	5	4
272	1990	gbr	1	4	5	4
273	1984	usa	1	1	1	
274	1984	usa	1	1	1	
275	1981	swe	1	1	1	
276	1977	pol	1	1	1	
277	1985	aus	1	1	1	
278	1987	bra	1	1	1	
279	1980	rsa	1	1	1	
280	1983	can	1	1	1	
281	1982	ita	1	2	1	
282	1983	can	1	2	1	
283	1985	aus	1	2	1	
284	1987	bra	1	2	1	
285	1975	usa	1	2	1	
286	1978	ned	1	2	1	
287	1980	rsa	1	2	1	
288	1977	rsa	1	2	1	
289	1985	usa	1	3	1	
290	1978	ned	1	3	1	
291	1989	kor	1	3	1	
292	1988	aus	1	3	1	
293	1978	ita	1	3	1	
294	1987	chn	1	3	1	
295	1986	ger	1	3	1	
296	1985	ita	1	3	1	
297	1989	kor	1	4	1	
298	1984	tun	1	4	1	
299	1980	aus	1	4	1	
300	1984	rus	1	4	1	
301	1984	usa	1	4	1	
302	1988	ita	1	4	1	
303	1980	aus	1	4	1	
304	1982	ukr	1	4	1	
305	1984	tun	1	5	1	

306	1985	pol	1	5	1
307	1980	aus	1	5	1
308	1988	ita	1	5	1
309	1986	fra	1	5	1
310	1982	ukr	1	5	1
311	1980	aus	1	5	1
312	1988	can	1	5	1
313	1987	pol	1	6	1
314	1984	rus	1	6	1
315	1985	gbr	1	6	1
316	1985	usa	1	6	1
317	1988	ita	1	6	1
318	1980	aus	1	6	1
319	1980	aus	1	6	1
320	1981	usa	1	6	1
321	1983	rsa	1	1	2
322	1977	ger	1	1	2
323	1985	gbr	1	1	2
324	1981	ger	1	1	2
325	1982	gbr	1	1	2
326	1985	gre	1	1	2
327	1987	jpn	1	1	2
328	1976	aus	1	1	2
329	1983	usa	1	2	2
330	1984	usa	1	2	2
331	1985	gbr	1	2	2
332	1984	rus	1	2	2
333	1982	aut	1	2	2
334	1983	rsa	1	2	2
335	1976	aus	1	2	2
336	1984	jpn	1	2	2
337	1984	usa	1	3	2
338	1983	usa	1	3	2
339	1982	aut	1	3	2
340	1984	rus	1	3	2
341	1980	rou	1	3	2
342	1983	gbr	1	3	2
343	1984	jpn	1	3	2
344	1979	gbr	1	3	2
345	1979	ukr	1	1	3
346	1981	usa	1	1	3
347	1988	rsa	1	1	3
348	1985	ita	1	1	3
349	1982	jpn	1	1	3
350	1985	isr	1	1	3
351	1983	aus	1	1	3
352	1985	ukr	1	1	3
353	1981	usa	1	2	3
354	1982	jpn	1	2	3
355	1983	aus	1	2	3
356	1985	ukr	1	2	3

357	1979	ukr	1	2	3
358	1985	bul	1	2	3
359	1981	rus	1	2	3
360	1985	nor	1	2	3
361	1982	jpn	1	3	3
362	1983	aus	1	3	3
363	1983	ita	1	3	3
364	1985	ita	1	3	3
365	1983	usa	1	3	3
366	1989	hun	1	3	3
367	1984	can	1	3	3
368	1987	rus	1	3	3
369	1980	rsa	1	1	4
370	1982	usa	1	1	4
371	1985	den	1	1	4
372	1986	ben	1	1	4
373	1983	ukr	1	1	4
374	1984	srb	1	1	4
375	1974	swe	1	1	4
376	1978	slo	1	1	4
377	1985	usa	1	2	4
378	1982	usa	1	2	4
379	1986	ven	1	2	4
380	1983	rsa	1	2	4
381	1982	ukr	1	2	4
382	1984	srb	1	2	4
383	1984	rus	1	2	4
384	1986	ken	1	2	4
385	1985	usa	1	3	4
386	1987	chn	1	3	4
387	1984	rus	1	3	4
388	1981	nzl	1	3	4
389	1983	jpn	1	3	4
390	1985	pol	1	3	4
391	1984	gre	1	3	4
392	1986	chn	1	3	4
393	1985	usa	1	3	5
394	1984	usa	1	3	5
395	1985	hun	1	3	5
396	1986	bra	1	3	5
397	1982	can	1	3	5
398	1979	hun	1	3	5
399	1981	ltu	1	3	5
400	1978	nzl	1	3	5
401	1985	usa	1	4	5
402	1984	usa	1	4	5
403	1986	ita	1	4	5
404	1984	tun	1	4	5
405	1985	hun	1	4	5
406	1984	gre	1	4	5
407	1983	gre	1	4	5

408	1979	hun	1	4	5
409	1985	aus	2	1	1
410	1977	swe	2	1	1
411	1979	ned	2	1	1
412	1983	ger	2	1	1
413	1985	usa	2	1	1
414	1983	aus	2	1	1
415	1982	fra	2	1	1
416	1982	usa	2	1	1
417	1985	aus	2	2	1
418	1979	ned	2	2	1
419	1983	ger	2	2	1
420	1982	usa	2	2	1
421	1989	can	2	2	1
422	1983	aus	2	2	1
423	1980	swe	2	2	1
424	1982	fra	2	2	1
425	1986	fra	2	3	1
426	1979	ger	2	3	1
427	1988	ita	2	3	1
428	1989	usa	2	3	1
429	1980	swe	2	3	1
430	1987	usa	2	3	1
431	1985	gbr	2	3	1
432	1983	pol	2	3	1
433	1986	fra	2	4	1
434	1983	pol	2	4	1
435	1982	jpn	2	4	1
436	1989	usa	2	4	1
437	1988	ita	2	4	1
438	1988	usa	2	4	1
439	1986	gbr	2	4	1
440	1983	aus	2	4	1
441	1988	usa	2	5	1
442	1986	fra	2	5	1
443	1985	usa	2	5	1
444	1984	esp	2	5	1
445	1985	fra	2	5	1
446	1982	jpn	2	5	1
447	1990	rsa	2	5	1
448	1990	aus	2	5	1
449	1988	usa	2	6	1
450	1981	sui	2	6	1
451	1982	jpn	2	6	1
452	1984	esp	2	6	1
453	1985	usa	2	6	1
454	1988	den	2	6	1
455	1985	chi	2	6	1
456	1986	fra	2	6	1
457	1985	usa	2	1	2
458	1985	blr	2	1	2

459	1985	aus	2	1	2
460	1990	chn	2	1	2
461	1982	jpn	2	1	2
462	1982	chn	2	1	2
463	1979	jpn	2	1	2
464	1982	ger	2	1	2
465	1982	usa	2	2	2
466	1986	fra	2	2	2
467	1982	jpn	2	2	2
468	1992	aus	2	2	2
469	1985	jpn	2	2	2
470	1986	ukr	2	2	2
471	1990	rus	2	2	2
472	1985	aus	2	2	2
473	1983	usa	2	3	2
474	1983	zim	2	3	2
475	1982	jpn	2	3	2
476	1987	fra	2	3	2
477	1985	jpn	2	3	2
478	1987	hun	2	3	2
479	1991	gbr	2	3	2
480	1987	ita	2	3	2
481	1987	usa	2	1	3
482	1985	aus	2	1	3
483	1982	usa	2	1	3
484	1981	aus	2	1	3
485	1981	ger	2	1	3
486	1976	nzl	2	1	3
487	1987	gbr	2	1	3
488	1987	swe	2	1	3
489	1985	aus	2	2	3
490	1982	usa	2	2	3
491	1988	ukr	2	2	3
492	1987	usa	2	2	3
493	1984	gbr	2	2	3
494	1981	aus	2	2	3
495	1987	gbr	2	2	3
496	1982	rus	2	2	3
497	1985	aus	2	3	3
498	1984	gbr	2	3	3
499	1984	usa	2	3	3
500	1988	rsa	2	3	3
501	1986	chn	2	3	3
502	1980	ger	2	3	3
503	1989	swe	2	3	3
504	1982	usa	2	3	3
505	1977	swe	2	1	4
506	1987	aus	2	1	4
507	1985	ned	2	1	4
508	1980	swe	2	1	4
509	1976	usa	2	1	4

510	1985	aut	2	1	4
511	1990	sin	2	1	4
512	1984	chn	2	1	4
513	1985	aus	2	2	4
514	1986	aus	2	2	4
515	1982	usa	2	2	4
516	1985	ned	2	2	4
517	1976	usa	2	2	4
518	1979	fra	2	2	4
519	1984	chn	2	2	4
520	1984	chn	2	2	4
521	1986	aus	2	3	4
522	1983	usa	2	3	4
523	1983	pol	2	3	4
524	1991	chn	2	3	4
525	1983	can	2	3	4
526	1981	jpn	2	3	4
527	1988	slo	2	3	4
528	1982	fra	2	3	4
529	1989	usa	2	3	5
530	1983	zim	2	3	5
531	1988	aus	2	3	5
532	1984	usa	2	3	5
533	1984	den	2	3	5
534	1982	aus	2	3	5
535	1983	arg	2	3	5
536	1987	can	2	3	5
537	1989	usa	2	4	5
538	1988	rus	2	4	5
539	1988	aus	2	4	5
540	1983	aus	2	4	5
541	1989	usa	2	4	5
542	1989	chn	2	4	5
543	1983	arg	2	4	5
544	1984	den	2	4	5
545	1983	ger	2	1	1
546	1977	swe	2	1	1
547	1992	aus	2	1	1
548	1979	ned	2	1	1
549	1990	gbr	2	1	1
550	1985	aus	2	1	1
551	1986	usa	2	1	1
552	1967	usa	2	1	1
553	1983	ger	2	2	1
554	1990	gbr	2	2	1
555	1985	aus	2	2	1
556	1986	usa	2	2	1
557	1987	usa	2	2	1
558	1990	ned	2	2	1
559	1988	den	2	2	1
560	1989	hun	2	2	1

561	1988	ita	2	3	1
562	1990	usa	2	3	1
563	1987	usa	2	3	1
564	1986	gbr	2	3	1
565	1985	chn	2	3	1
566	1985	chn	2	3	1
567	1988	hun	2	3	1
568	1989	hun	2	3	1
569	1988	ita	2	4	1
570	1986	gbr	2	4	1
571	1989	gbr	2	4	1
572	1990	usa	2	4	1
573	1987	fra	2	4	1
574	1982	rou	2	4	1
575	1990	fra	2	4	1
576	1988	den	2	4	1
577	1988	den	2	5	1
578	1986	gbr	2	5	1
579	1987	ita	2	5	1
580	1989	gbr	2	5	1
581	1982	rou	2	5	1
582	1984	esp	2	5	1
583	1990	fra	2	5	1
584	1990	rsa	2	5	1
585	1987	ita	2	6	1
586	1988	den	2	6	1
587	1982	rou	2	6	1
588	1985	chi	2	6	1
589	1984	esp	2	6	1
590	1990	rsa	2	6	1
591	1985	aus	2	6	1
592	1992	usa	2	6	1
593	1990	chn	2	1	2
594	1984	ger	2	1	2
595	1987	chn	2	1	2
596	1990	rus	2	1	2
597	1985	blr	2	1	2
598	1984	aus	2	1	2
599	1992	aus	2	1	2
600	1975	bra	2	1	2
601	1987	gbr	2	2	2
602	1990	rus	2	2	2
603	1992	aus	2	2	2
604	1990	jpn	2	2	2
605	1990	chn	2	2	2
606	1986	usa	2	2	2
607	1991	gbr	2	2	2
608	1983	zim	2	2	2
609	1983	zim	2	3	2
610	1990	rus	2	3	2
611	1992	usa	2	3	2

612	1987	gbr	2	3	2
613	1991	gbr	2	3	2
614	1993	usa	2	3	2
615	1990	fra	2	3	2
616	1984	jpn	2	3	2
617	1992	rus	2	1	3
618	1987	usa	2	1	3
619	1984	aus	2	1	3
620	1988	ned	2	1	3
621	1983	can	2	1	3
622	1991	usa	2	1	3
623	1993	can	2	1	3
624	1981	aus	2	1	3
625	1987	usa	2	2	3
626	1992	rus	2	2	3
627	1991	usa	2	2	3
628	1984	aus	2	2	3
629	1983	can	2	2	3
630	1989	den	2	2	3
631	1986	aut	2	2	3
632	1983	ger	2	2	3
633	1987	srb	2	3	3
634	1983	can	2	3	3
635	1986	aut	2	3	3
636	1987	usa	2	3	3
637	1988	jpn	2	3	3
638	1987	jpn	2	3	3
639	1989	can	2	3	3
640	1988	swe	2	3	3
641	1986	aus	2	1	4
642	1984	chn	2	1	4
643	1988	nor	2	1	4
644	1977	swe	2	1	4
645	1979	ned	2	1	4
646	1993	swe	2	1	4
647	1979	fra	2	1	4
648	1989	bra	2	1	4
649	1993	swe	2	2	4
650	1986	aus	2	2	4
651	1991	chn	2	2	4
652	1982	fra	2	2	4
653	1988	bra	2	2	4
654	1987	usa	2	2	4
655	1988	nor	2	2	4
656	1979	ned	2	2	4
657	1986	aus	2	3	4
658	1989	chn	2	3	4
659	1989	hun	2	3	4
660	1984	usa	2	3	4
661	1991	chn	2	3	4
662	1982	fra	2	3	4

663	1983	can	2	3	4
664	1991	aus	2	3	4
665	1989	usa	2	3	5
666	1988	aus	2	3	5
667	1989	hun	2	3	5
668	1983	zim	2	3	5
669	1984	den	2	3	5
670	1989	gbr	2	3	5
671	1989	hun	2	3	5
672	1989	fra	2	3	5
673	1989	hun	2	4	5
674	1983	zim	2	4	5
675	1988	aus	2	4	5
676	1989	gbr	2	4	5
677	1992	usa	2	4	5
678	1987	usa	2	4	5
679	1989	hun	2	4	5
680	1980	can	2	4	5
681	1987	bra	1	1	1
682	1981	fra	1	1	1
683	1985	fra	1	1	1
684	1983	cro	1	1	1
685	1984	usa	1	1	1
686	1988	usa	1	1	1
687	1983	tri	1	1	1
688	1981	swe	1	1	1
689	1987	bra	1	2	1
690	1983	fra	1	2	1
691	1981	fra	1	2	1
692	1983	can	1	2	1
693	1987	usa	1	2	1
694	1981	swe	1	2	1
695	1983	rsa	1	2	1
696	1984	bra	1	2	1
697	1986	ger	1	3	1
698	1985	usa	1	3	1
699	1991	rus	1	3	1
700	1987	jpn	1	3	1
701	1988	aus	1	3	1
702	1987	rsa	1	3	1
703	1988	ned	1	3	1
704	1988	rus	1	3	1
705	1986	ger	1	4	1
706	1984	tun	1	4	1
707	1987	chn	1	4	1
708	1984	usa	1	4	1
709	1988	den	1	4	1
710	1988	hun	1	4	1
711	1988	can	1	4	1
712	1985	gbr	1	4	1
713	1987	chn	1	5	1

714	1984	tun	1	5	1
715	1988	can	1	5	1
716	1988	iita	1	5	1
717	1985	gbr	1	5	1
718	1984	usa	1	5	1
719	1983	esp	1	5	1
720	1984	rus	1	5	1
721	1984	tun	1	6	1
722	1988	can	1	6	1
723	1991	chn	1	6	1
724	1988	ita	1	6	1
725	1987	chn	1	6	1
726	1985	gbr	1	6	1
727	1983	esp	1	6	1
728	1986	ita	1	6	1
729	1985	gbr	1	1	2
730	1987	jpn	1	1	2
731	1983	rsa	1	1	2
732	1986	esp	1	1	2
733	1985	fra	1	1	2
734	1984	ger	1	1	2
735	1985	gre	1	1	2
736	1985	ita	1	1	2
737	1987	jpn	1	2	2
738	1984	ger	1	2	2
739	1986	esp	1	2	2
740	1990	jpn	1	2	2
741	1985	gbr	1	2	2
742	1984	rus	1	2	2
743	1985	usa	1	2	2
744	1985	gre	1	2	2
745	1983	usa	1	3	2
746	1990	jpn	1	3	2
747	1984	usa	1	3	2
748	1984	rus	1	3	2
749	1986	esp	1	3	2
750	1983	rus	1	3	2
751	1991	pol	1	3	2
752	1982	rsa	1	3	2
753	1988	rsa	1	1	3
754	1987	bra	1	1	3
755	1982	usa	1	1	3
756	1986	ger	1	1	3
757	1983	aus	1	1	3
758	1983	slo	1	1	3
759	1979	slo	1	1	3
760	1986	bra	1	1	3
761	1983	aus	1	2	3
762	1981	fra	1	2	3
763	1988	rsa	1	2	3
764	1983	usa	1	2	3

765	1984	ukr	1	2	3
766	1989	ltu	1	2	3
767	1986	ger	1	2	3
768	1984	bra	1	2	3
769	1989	hun	1	3	3
770	1983	usa	1	3	3
771	1989	ltu	1	3	3
772	1985	aus	1	3	3
773	1983	aus	1	3	3
774	1989	ita	1	3	3
775	1984	bra	1	3	3
776	1983	ita	1	3	3
777	1984	srb	1	1	4
778	1985	aus	1	1	4
779	1988	esp	1	1	4
780	1985	den	1	1	4
781	1980	bra	1	1	4
782	1986	ken	1	1	4
783	1986	ven	1	1	4
784	1983	cro	1	1	4
785	1985	usa	1	2	4
786	1984	srb	1	2	4
787	1988	esp	1	2	4
788	1986	ven	1	2	4
789	1987	aus	1	2	4
790	1986	ken	1	2	4
791	1987	usa	1	2	4
792	1982	bra	1	2	4
793	1985	usa	1	3	4
794	1985	pol	1	3	4
795	1984	jpn	1	3	4
796	1984	bra	1	3	4
797	1989	usa	1	3	4
798	1989	aut	1	3	4
799	1990	rsa	1	3	4
800	1987	gbr	1	3	4
801	1984	usa	1	3	5
802	1985	hun	1	3	5
803	1983	usa	1	3	5
804	1986	bra	1	3	5
805	1986	aus	1	3	5
806	1983	gbr	1	3	5
807	1985	jpn	1	3	5
808	1988	hun	1	3	5
809	1984	usa	1	4	5
810	1989	usa	1	4	5
811	1985	hun	1	4	5
812	1986	bra	1	4	5
813	1988	hun	1	4	5
814	1987	isr	1	4	5
815	1986	ita	1	4	5

816	1988	gbr	1	4	5
817	1987	bra	1	1	1
818	1990	ita	1	1	1
819	1983	fra	1	1	1
820	1988	usa	1	1	1
821	1991	bra	1	1	1
822	1985	hun	1	1	1
823	1983	tri	1	1	1
824	1987	rsa	1	1	1
825	1991	aus	1	2	1
826	1983	can	1	2	1
827	1987	fra	1	2	1
828	1987	bra	1	2	1
829	1984	fra	1	2	1
830	1988	usa	1	2	1
831	1990	ita	1	2	1
832	1988	ned	1	2	1
833	1984	usa	1	3	1
834	1985	usa	1	3	1
835	1986	ger	1	3	1
836	1989	kor	1	3	1
837	1992	fra	1	3	1
838	1988	rus	1	3	1
839	1984	sui	1	3	1
840	1991	rus	1	3	1
841	1989	kor	1	4	1
842	1991	chn	1	4	1
843	1986	ger	1	4	1
844	1984	usa	1	4	1
845	1988	can	1	4	1
846	1992	fra	1	4	1
847	1984	tun	1	4	1
848	1986	fra	1	4	1
849	1991	chn	1	5	1
850	1988	can	1	5	1
851	1988	hun	1	5	1
852	1984	tun	1	5	1
853	1990	far	1	5	1
854	1988	usa	1	5	1
855	1984	usa	1	5	1
856	1986	fra	1	5	1
857	1991	chn	1	6	1
858	1988	can	1	6	1
859	1988	hun	1	6	1
860	1990	far	1	6	1
861	1988	usa	1	6	1
862	1984	usa	1	6	1
863	1986	ita	1	6	1
864	1990	jpn	1	6	1
865	1985	gbr	1	1	2
866	1985	fra	1	1	2

867	1983	rsa	1	1	2
868	1986	esp	1	1	2
869	1985	usa	1	1	2
870	1986	usa	1	1	2
871	1987	isr	1	1	2
872	1983	sui	1	1	2
873	1985	fra	1	2	2
874	1988	fra	1	2	2
875	1990	jpn	1	2	2
876	1986	usa	1	2	2
877	1985	usa	1	2	2
878	1985	gbr	1	2	2
879	1984	ger	1	2	2
880	1991	nzl	1	2	2
881	1984	usa	1	3	2
882	1990	jpn	1	3	2
883	1989	usa	1	3	2
884	1993	chn	1	3	2
885	1991	pol	1	3	2
886	1983	rus	1	3	2
887	1985	ita	1	3	2
888	1987	jpn	1	3	2
889	1987	bra	1	1	3
890	1988	ita	1	1	3
891	1988	rsa	1	1	3
892	1986	ger	1	1	3
893	1985	nor	1	1	3
894	1982	usa	1	1	3
895	1986	ned	1	1	3
896	1988	slo	1	1	3
897	1985	nor	1	2	3
898	1988	ita	1	2	3
899	1988	rsa	1	2	3
900	1982	jpn	1	2	3
901	1983	aus	1	2	3
902	1989	hun	1	2	3
903	1989	ltu	1	2	3
904	1982	usa	1	2	3
905	1989	hun	1	3	3
906	1982	jpn	1	3	3
907	1992	ger	1	3	3
908	1983	usa	1	3	3
909	1988	gbr	1	3	3
910	1989	ltu	1	3	3
911	1990	kor	1	3	3
912	1990	gbr	1	3	3
913	1987	bra	1	1	4
914	1985	aus	1	1	4
915	1979	aus	1	1	4
916	1981	fra	1	1	4
917	1990	fra	1	1	4

918	1987	ger	1	1	4
919	1986	ken	1	1	4
920	1992	ukr	1	1	4
921	1985	usa	1	2	4
922	1989	pol	1	2	4
923	1987	usa	1	2	4
924	1986	ken	1	2	4
925	1985	jpn	1	2	4
926	1983	rus	1	2	4
927	1988	ned	1	2	4
928	1979	aus	1	2	4
929	1985	usa	1	3	4
930	1984	jpn	1	3	4
931	1987	chn	1	3	4
932	1986	chn	1	3	4
933	1992	rsa	1	3	4
934	1985	pol	1	3	4
935	1989	aut	1	3	4
936	1993	hun	1	3	4
937	1984	usa	1	3	5
938	1985	usa	1	3	5
939	1985	hun	1	3	5
940	1983	gbr	1	3	5
941	1982	aut	1	3	5
942	1986	bra	1	3	5
943	1992	aus	1	3	5
944	1990	jpn	1	3	5
945	1984	usa	1	4	5
946	1989	usa	1	4	5
947	1990	jpn	1	4	5
948	1994	chn	1	4	5
949	1984	gre	1	4	5
950	1988	hun	1	4	5
951	1992	chn	1	4	5
952	1991	gbr	1	4	5
953	1977	swe	2	1	1
954	1990	ned	2	1	1
955	1979	ned	2	1	1
956	1990	gbr	2	1	1
957	1985	blr	2	1	1
958	1987	den	2	1	1
959	1990	bah	2	1	1
960	1987	usa	2	1	1
961	1987	den	2	2	1
962	1985	blr	2	2	1
963	1990	ned	2	2	1
964	1990	gbr	2	2	1
965	1987	ned	2	2	1
966	1987	aus	2	2	1
967	1987	usa	2	2	1
968	1982	usa	2	2	1

969	1988	ita	2	3	1
970	1990	aus	2	3	1
971	1989	fra	2	3	1
972	1993	swe	2	3	1
973	1989	aus	2	3	1
974	1990	usa	2	3	1
975	1987	ned	2	3	1
976	1994	ger	2	3	1
977	1988	ita	2	4	1
978	1989	gbr	2	4	1
979	1989	fra	2	4	1
980	1990	aus	2	4	1
981	1988	den	2	4	1
982	1987	nzl	2	4	1
983	1989	usa	2	4	1
984	1989	esp	2	4	1
985	1989	gbr	2	5	1
986	1988	den	2	5	1
987	1988	usa	2	5	1
988	1992	usa	2	5	1
989	1993	hun	2	5	1
990	1992	aus	2	5	1
991	1990	rsa	2	5	1
992	1987	nzl	2	5	1
993	1988	den	2	6	1
994	1988	usa	2	6	1
995	1994	chn	2	6	1
996	1985	chi	2	6	1
997	1985	aus	2	6	1
998	1990	rsa	2	6	1
999	1984	esp	2	6	1
1000	1995	chn	2	6	1
1001	1990	rus	2	1	2
1002	1984	jpn	2	1	2
1003	1995	usa	2	1	2
1004	1987	chn	2	1	2
1005	1992	aus	2	1	2
1006	1987	can	2	1	2
1007	1985	blr	2	1	2
1008	1985	esp	2	1	2
1009	1990	chn	2	2	2
1010	1990	rus	2	2	2
1011	1982	usa	2	2	2
1012	1992	aus	2	2	2
1013	1984	jpn	2	2	2
1014	1990	aus	2	2	2
1015	1991	gbr	2	2	2
1016	1993	den	2	2	2
1017	1995	usa	2	3	2
1018	19990	aus	2	3	2
1019	1993	ned	2	3	2

1020	1994	ukr	2	3	2
1021	1992	usa	2	3	2
1022	1988	aus	2	3	2
1023	1991	gbr	2	3	2
1024	1990	fra	2	3	2
1025	1987	usa	2	1	3
1026	1992	rus	2	1	3
1027	1987	usa	2	1	3
1028	1992	aus	2	1	3
1029	1988	swe	2	1	3
1030	1985	aus	2	1	3
1031	1988	ned	2	1	3
1032	1987	swe	2	1	3
1033	1987	usa	2	2	3
1034	1985	aus	2	2	3
1035	1988	chn	2	2	3
1036	1992	rus	2	2	3
1037	1989	chn	2	2	3
1038	1989	den	2	2	3
1039	1988	can	2	2	3
1040	1988	ned	2	2	3
1041	1987	usa	2	3	3
1042	1992	rus	2	3	3
1043	1989	can	2	3	3
1044	1989	chn	2	3	3
1045	1988	jpn	2	3	3
1046	1987	srb	2	3	3
1047	1989	den	2	3	3
1048	1983	can	2	3	3
1049	1985	ned	2	1	4
1050	1977	swe	2	1	4
1051	1992	fra	2	1	4
1052	1989	chn	2	1	4
1053	1993	swe	2	1	4
1054	1986	jpn	2	1	4
1055	1987	usa	2	1	4
1056	1986	aus	2	1	4
1057	1987	usa	2	2	4
1058	1987	aus	2	2	4
1059	1989	chn	2	2	4
1060	1993	swe	2	2	4
1061	1991	gbr	2	2	4
1062	1989	chn	2	2	4
1063	1986	aus	2	2	4
1064	1990	gbr	2	2	4
1065	1991	chn	2	3	4
1066	1991	gbr	2	3	4
1067	1989	chn	2	3	4
1068	1990	jpn	2	3	4
1069	1988	aus	2	3	4
1070	1989	hun	2	3	4

1071	1990 gbr	2	3	4
1072	1986 aus	2	3	4
1073	1996 chn	2	3	5
1074	1987 aus	2	3	5
1075	1989 usa	2	3	5
1076	1988 aus	2	3	5
1077	1991 usa	2	3	5
1078	1989 hun	2	3	5
1079	1989 gbr	2	3	5
1080	1987 can	2	3	5
1081	1992 usa	2	4	5
1082	1989 gbr	2	4	5
1083	1988 aus	2	4	5
1084	1990 esp	2	4	5
1085	1996 chn	2	4	5
1086	1994 chn	2	4	5
1087	1993 cze	2	4	5
1088	1991 usa	2	4	5
1089	1987 bra	1	1	1
1090	1992 rus	1	1	1
1091	1983 tri	1	1	1
1092	1988 usa	1	1	1
1093	1990 fra	1	1	1
1094	1981 usa	1	1	1
1095	1980 rsa	1	1	1
1096	1981 fra	1	1	1
1097	1991 aus	1	2	1
1098	1989 usa	1	2	1
1099	1988 usa	1	2	1
1100	1994 aus	1	2	1
1101	1992 rus	1	2	1
1102	1991 bra	1	2	1
1103	1984 fra	1	2	1
1104	1990 ita	1	2	1
1105	1992 fra	1	3	1
1106	1989 usa	1	3	1
1107	1991 rus	1	3	1
1108	1984 usa	1	3	1
1109	1994 jpn	1	3	1
1110	1988 gbr	1	3	1
1111	1994 aus	1	3	1
1112	1991 aus	1	3	1
1113	1991 chn	1	4	1
1114	1994 jpn	1	4	1
1115	1991 usa	1	4	1
1116	1988 can	1	4	1
1117	1995 gbr	1	4	1
1118	1992 rsa	1	4	1
1119	1995 aus	1	4	1
1120	1995 chn	1	4	1
1121	1991 chn	1	5	1

1122	1991	usa	1	5	1
1123	1988	can	1	5	1
1124	1991	usa	1	5	1
1125	1995	aus	1	5	1
1126	1994	ita	1	5	1
1127	1990	far	1	5	1
1128	1984	tun	1	5	1
1129	1991	chn	1	6	1
1130	1988	can	1	6	1
1131	1994	ita	1	6	1
1132	1991	usa	1	6	1
1133	1991	usa	1	6	1
1134	1995	aus	1	6	1
1135	1990	far	1	6	1
1136	1987	gbr	1	6	1
1137	1985	fra	1	1	2
1138	1988	fra	1	1	2
1139	1985	usa	1	1	2
1140	1986	esp	1	1	2
1141	1986	chn	1	1	2
1142	1985	bra	1	1	2
1143	1987	isr	1	1	2
1144	1991	isr	1	1	2
1145	1985	usa	1	2	2
1146	1985	usa	1	2	2
1147	1988	fra	1	2	2
1148	1990	jpn	1	2	2
1149	1985	fra	1	2	2
1150	1986	aus	1	2	2
1151	1994	jpn	1	2	2
1152	1991	nzl	1	2	2
1153	1984	usa	1	3	2
1154	1991	pol	1	3	2
1155	1989	usa	1	3	2
1156	1990	jpn	1	3	2
1157	1994	jpn	1	3	2
1158	1992	gbr	1	3	2
1159	1995	chn	1	3	2
1160	1992	hun	1	3	2
1161	1988	rsa	1	1	3
1162	1985	aus	1	1	3
1163	1989	rsa	1	1	3
1164	1988	slo	1	1	3
1165	1986	bra	1	1	3
1166	1987	nzl	1	1	3
1167	1995	swe	1	1	3
1168	1989	ita	1	1	3
1169	1985	aus	1	2	3
1170	1998	rsa	1	2	3
1171	1985	bra	1	2	3
1172	1988	slo	1	2	3

1173	1988	ita	1	2	3
1174	1982	jpn	1	2	3
1175	1993	usa	1	2	3
1176	1993	usa	1	2	3
1177	1989	hun	1	3	3
1178	1990	ger	1	3	3
1179	1993	fin	1	3	3
1180	1990	gbr	1	3	3
1181	1988	gbr	1	3	3
1182	1991	rus	1	3	3
1183	1994	jpn	1	3	3
1184	1989	jpn	1	3	3
1185	1987	bra	1	1	4
1186	1988	usa	1	1	4
1187	1981	fra	1	1	4
1188	1980	bra	1	1	4
1189	1992	ukr	1	1	4
1190	1990	blr	1	1	4
1191	1987	ger	1	1	4
1192	1990	fra	1	1	4
1193	1992	rsa	1	2	4
1194	1985	hun	1	2	4
1195	1989	pol	1	2	4
1196	1987	ger	1	2	4
1197	1972	rus	1	2	4
1198	1984	usa	1	2	4
1199	1991	ita	1	2	4
1200	1990	blr	1	2	4
1201	1992	rsa	1	3	4
1202	1985	pol	1	3	4
1203	1987	chn	1	3	4
1204	1986	chn	1	3	4
1205	1991	usa	1	3	4
1206	1984	rus	1	3	4
1207	1989	usa	1	3	4
1208	1991	bra	1	3	4
1209	1984	usa	1	3	5
1210	1994	jpn	1	3	5
1211	1986	bra	1	3	5
1212		chn	1	3	5
1213	1985	hun	1	3	5
1214	1992	aus	1	3	5
1215	1994	jpn	1	3	5
1216	1986	swe	1	3	5
1217	1994	jpn	1	4	5
1218	1994	usa	1	4	5
1219	1986	bra	1	4	5
1220	1989	usa	1	4	5
1221	1994	jpn	1	4	5
1222	1988	hun	1	4	5
1223	1993	gbr	1	4	5

1224	1991	aus	1	4	5
1225	1990	ned	2	1	1
1226	1992	aus	2	1	1
1227	1990	gbr	2	1	1
1228	1993	swe	2	1	1
1229	1994	aus	2	1	1
1230	1987	den	2	1	1
1231	1996	usa	2	1	1
1232	1984	ger	2	1	1
1233	1992	aus	2	2	1
1234	1993	swe	2	2	1
1235	1990	ned	2	2	1
1236	1995	usa	2	2	1
1237	1987	ned	2	2	1
1238	1983	ger	2	2	1
1239	1993	chn	2	2	1
1240	1991	usa	2	2	1
1241	1995	usa	2	3	1
1242	1988	ita	2	3	1
1243	1989	fra	2	3	1
1244	1993	swe	2	3	1
1245	1989	sesp	2	3	1
1246	1990	aus	2	3	1
1247	1991	usa	2	3	1
1248	1995	fra	2	3	1
1249	1997	usa	2	4	1
1250	1989	esp	2	4	1
1251	1987	nzl	2	4	1
1252	1990	gbr	2	4	1
1253	1993	hun	2	4	1
1254	1991	ven	2	4	1
1255	1989	fra	2	4	1
1256	1990	aus	2	4	1
1257	1997	usa	2	5	1
1258	1988	den	2	5	1
1259	1987	nzl	2	5	1
1260	1993	hun	2	5	1
1261	1990	esp	2	5	1
1262	1992	usa	2	5	1
1263	1991	ven	2	5	1
1264	1991	ita	2	5	1
1265	1997	usa	2	6	1
1266	1988	den	2	6	1
1267	1987	nzl	2	6	1
1268	1990	esp	2	6	1
1269	1998	chn	2	6	1
1270	1985	chi	2	6	1
1271	1993	hun	2	6	1
1272	1992	usa	2	6	1
1273	1990	chn	2	1	2
1274	1996	chn	2	1	2

1275	1984	jpn	2	1	2
1276	1991	bra	2	1	2
1277	1985	esp	2	1	2
1278	1990	gbr	2	1	2
1279	1993	usa	2	1	2
1280	1995	gbr	2	1	2
1281	1995	ltu	2	2	2
1282	1992	rus	2	2	2
1283	1984	usa	2	2	2
1284	1993	den	2	2	2
1285	1996	usa	2	2	2
1286	1991	ukr	2	2	2
1287	1994	esp	2	2	2
1288	1990	swe	2	2	2
1289	1995	usa	2	3	2
1290	1990	aus	2	3	2
1291	1991	can	2	3	2
1292	1994	ukr	2	3	2
1293	1993	usa	2	3	2
1294	1989	hun	2	3	2
1295	1993	can	2	3	2
1296	1998	rus	2	3	2
1297	1992	rus	2	1	3
1298	1997	ltu	2	1	3
1299	1987	usa	2	1	3
1300	1992	usa	2	1	3
1301	1988	swe	2	1	3
1302	1989	den	2	1	3
1303	1988	ned	2	1	3
1304	1986	cze	2	1	3
1305	1997	usa	2	2	3
1306	1992	aus	2	2	3
1307	1987	jpn	2	2	3
1308	1989	usa	2	2	3
1309	1992	chn	2	2	3
1310	1998	cze	2	2	3
1311	1994	ukr	2	2	3
1312	1988	aus	2	2	3
1313	1992	rus	2	3	3
1314	1989	den	2	3	3
1315	1990	usa	2	3	3
1316	1988	jpn	2	3	3
1317	1998	ukr	2	3	3
1318	1994	esp	2	3	3
1319		aus	2	3	3
1320	1989	can	2	3	3
1321	1987	den	2	1	4
1322	1989	chn	2	1	4
1323	1990	ned	2	1	4
1324	1990	gbr	2	1	4
1325	1985	ned	2	1	4

1326	1992	fra	2	1	4
1327	1995	egy	2	1	4
1328	1987	usa	2	1	4
1329	1993	swe	2	2	4
1330	1987	aus	2	2	4
1331	1987	usa	2	2	4
1332	1987	den	2	2	4
1333	1993	can	2	2	4
1334	1990	ita	2	2	4
1335	1996	can	2	2	4
1336	1989	usa	2	2	4
1337	1989	chn	2	3	4
1338	1990	esp	2	3	4
1339	1989	hun	2	3	4
1340	1990	jpn	2	3	4
1341	1989	hun	2	3	4
1342	1991	chn	2	3	4
1343	1991	usa	2	3	4
1344	1994	esp	2	3	4
1345	1989	hun	2	3	5
1346	1987	aus	2	3	5
1347	1990	esp	2	3	5
1348	1996	chn	2	3	5
1349	1991	usa	2	3	5
1350	1989	hun	2	3	5
1351	1992	gbr	2	3	5
1352	1995	gbr	2	3	5
1353	1989	hun	2	4	5
1354	1990	esp	2	4	5
1355	1992	usa	2	4	5
1356	1993	usa	2	4	5
1357		gbr	2	4	5
1358	1989	hun	2	4	5
1359	1996	chn	2	4	5
1360	1994	jpn	2	4	5
1361	1990	fra	1	1	1
1362	1988	usa	1	1	1
1363	1989	bra	1	1	1
1364	1992	rus	1	1	1
1365	1992	ukr	1	1	1
1366	1990	ita	1	1	1
1367	1993	gre	1	1	1
1368	1994	gbr	1	1	1
1369	1993	chn	1	2	1
1370	1994	aus	1	2	1
1371	1990	arg	1	2	1
1372	1995	can	1	2	1
1373	1991	bra	1	2	1
1374	1988	rus	1	2	1
1375	1988	usa	1	2	1
1376	1988	bel	1	2	1

1377	1995	gbr	1	3	1
1378	1991	chn	1	3	1
1379	1986	ger	1	3	1
1380	1984	usa	1	3	1
1381	1988	ned	1	3	1
1382	1992	rsa	1	3	1
1383	1995	rus	1	3	1
1384	1994	aus	1	3	1
1385	1991	chn	1	4	1
1386	1995	gbr	1	4	1
1387	1988	can	1	4	1
1388	1991	usa	1	4	1
1389	1992	hun	1	4	1
1390	1996	pol	1	4	1
1391	1989	ger	1	4	1
1392	1991	usa	1	4	1
1393	1991	chn	1	5	1
1394	1994	ita	1	5	1
1395	1996	aus	1	5	1
1396	1991	usa	1	5	1
1397	1996	nor	1	5	1
1398	1996	pol	1	5	1
1399	1994	gbr	1	5	1
1400	1991	usa	1	5	1
1401	1994	ita	1	6	1
1402	1991	usa	1	6	1
1403	1988	can	1	6	1
1404	1996	egy	1	6	1
1405	1994	gbr	1	6	1
1406	1991	usa	1	6	1
1407	1996	ukr	1	6	1
1408	1991	chn	1	6	1
1409	1985	fra	1	1	2
1410	1985	usa	1	1	2
1411	1991	aus	1	1	2
1412	1993	aus	1	1	2
1413	1992	rus	1	1	2
1414	1992	nor	1	1	2
1415	1985	gbr	1	1	2
1416	1985	usa	1	1	2
1417	1993	aus	1	2	2
1418	1985	fra	1	2	2
1419	1985	usa	1	2	2
1420	1995	chn	1	2	2
1421	1990	gbr	1	2	2
1422	1990	jpn	1	2	2
1423	1996	rus	1	2	2
1424	1985	gbr	1	2	2
1425	1993	aus	1	3	2
1426	1991	pol	1	3	2
1427	1996	rus	1	3	2

1428	1990	jpn	1	3	2
1429	1995	usa	1	3	2
1430	1995	chn	1	3	2
1431	1989	usa	1	3	2
1432	1997	chn	1	3	2
1433	1994	gbr	1	1	3
1434	1988	rsa	1	1	3
1435	1993	usa	1	1	3
1436	1987	bra	1	1	3
1437	1988	slo	1	1	3
1438	1989	ltu	1	1	3
1439	1987	nzl	1	1	3
1440	1990	srb	1	1	3
1441	1994	gbr	1	2	3
1442	1988	rsa	1	2	3
1443	1994	gbr	1	2	3
1444	1995	kaz	1	2	3
1445	1994	aus	1	2	3
1446	1989	ltu	1	2	3
1447	1995	rus	1	2	3
1448	1986	ger	1	2	3
1449	1990	ger	1	3	3
1450	1993	usa	1	3	3
1451	1989	hun	1	3	3
1452	1990	gbr	1	3	3
1453	1992	hjpn	1	3	3
1454	1995	kaz	1	3	3
1455	1997	rus	1	3	3
1456	1993	chn	1	3	3
1457	1990	fra	1	1	4
1458	1980	bra	1	1	4
1459	1985	hun	1	1	4
1460	1989	pol	1	1	4
1461	1992	ukr	1	1	4
1462	1987	bra	1	1	4
1463	1995	sin	1	1	4
1464	1994	gbr	1	1	4
1465	1992	rsa	1	2	4
1466	1985	hun	1	2	4
1467	1995	sin	1	2	4
1468	1991	usa	1	2	4
1469	1992	fra	1	2	4
1470	1989	pol	1	2	4
1471	1985	pol	1	2	4
1472	1999	chn	1	2	4
1473	1985	hun	1	3	4
1474	1992	rsa	1	3	4
1475	1994	pol	1	3	4
1476	1995	jpn	1	3	4
1477	1993	den	1	3	4
1478	1994	jpn	1	3	4

1479	1994	bel	1	3	4
1480	1991	usa	1	3	4
1481	1984	usa	1	3	5
1482	1986	bra	1	3	5
1483	1994	chn	1	3	5
1484	1993	gbr	1	3	5
1485	1989	usa	1	3	5
1486	1992	pol	1	3	5
1487	1991	bra	1	3	5
1488	1986	swe	1	3	5
1489	1994	jpn	1	4	5
1490	1988	hun	1	4	5
1491	1994	usa	1	4	5
1492	1989	usa	1	4	5
1493	1994	ger	1	4	5
1494	1993	gbr	1	4	5
1495	1991	gbr	1	4	5
1496	1992	chn	1	4	5
1497	1992	aus	2	1	1
1498	1990	ned	2	1	1
1499	1993	swe	2	1	1
1500	1994	aus	2	1	1
1501	1994	can	2	1	1
1502	1990	bah	2	1	1
1503	1990	gbr	2	1	1
1504	1996	usa	2	1	1
1505	1994	aus	2	2	1
1506	1993	swe	2	2	1
1507	1994	aus	2	2	1
1508	1990	ned	2	2	1
1509	1987	ned	2	2	1
1510	1996	usa	2	2	1
1511	1995	usa	2	2	1
1512	1997	chn	2	2	1
1513	1997	usa	2	3	1
1514	1988	ita	2	3	1
1515	1995	usa	2	3	1
1516	1991	rus	2	3	1
1517	1989	hun	2	3	1
1518	1997	chn	2	3	1
1519	1994	aus	2	3	1
1520	1987	ned	2	3	1
1521	1997	usa	2	4	1
1522	1993	ned	2	4	1
1523	1993	aus	2	4	1
1524	1990	gbr	2	4	1
1525	1987	nzl	2	4	1
1526	1989	esp	2	4	1
1527	1996	ita	2	4	1
1528	1993	hun	2	4	1
1529	1997	usa	2	5	1

1530	1987	nzl	2	5	1
1531	1990	gbr	2	5	1
1532	1993	aus	2	5	1
1533	1988	den	2	5	1
1534	1993	hun	2	5	1
1535	1994	ger	2	5	1
1536	1993	ned	2	5	1
1537	1997	usa	2	6	1
1538	1987	nzl	2	6	1
1539	1993	hun	2	6	1
1540	1988	den	2	6	1
1541	1993	aus	2	6	1
1542	1993	ned	2	6	1
1543	1985	chi	2	6	1
1544	1992	ita	2	6	1
1545	1996	chn	2	1	2
1546	1991	bra	2	1	2
1547	1996	chn	2	1	2
1548	1992	aus	2	1	2
1549	1996	den	2	1	2
1550	1994	aus	2	1	2
1551	1995	gbr	2	1	2
1552	1992	gre	2	1	2
1553	1992	aus	2	2	2
1554	1994	aus	2	2	2
1555	1996	den	2	2	2
1556	1996	chn	2	2	2
1557	1995	usa	2	2	2
1558	1990	rus	2	2	2
1559	1995	gbr	2	2	2
1560	1997	usa	2	2	2
1561	1992	aus	2	3	2
1562	1995	usa	2	3	2
1563	1989	hun	2	3	2
1564	1998	rus	2	3	2
1565	1986	ger	2	3	2
1566	1991	can	2	3	2
1567	1991	can	2	3	2
1568	1995	isl	2	3	2
1569	1988	swe	2	1	3
1570	1988	jam	2	1	3
1571	1992	rus	2	1	3
1572	1997	ltu	2	1	3
1573	1987	usa	2	1	3
1574	1994	chn	2	1	3
1575	1991	isl	2	1	3
1576	1990	ukr	2	1	3
1577	1992	rus	2	2	3
1578	1997	ltu	2	2	3
1579	1988	jam	2	2	3
1580	1996	jpn	2	2	3

1581	1993	chn	2	2	3
1582	1991	isl	2	2	3
1583	1988	swe	2	2	3
1584	1997	ita	2	2	3
1585	1996	jpn	2	3	3
1586	1990	usa	2	3	3
1587	1988	esp	2	3	3
1588	1989	den	2	3	3
1589	1993	chn	2	3	3
1590	1988	jpn	2	3	3
1591	1992	rus	2	3	3
1592	1994	can	2	3	3
1593	1993	swe	2	1	4
1594	1987	den	2	1	4
1595	1989	chn	2	1	4
1596	1985	ned	2	1	4
1597	1995	egy	2	1	4
1598	1990	gbr	2	1	4
1599	1990	bah	2	1	4
1600	1990	pol	2	1	4
1601	1993	swe	2	2	4
1602	1987	den	2	2	4
1603	1989	chn	2	2	4
1604	1994	aus	2	2	4
1605	1993	can	2	2	4
1606	1998	chn	2	2	4
1607	1995	ger	2	2	4
1608	1996	can	2	2	4
1609	1990	jpn	2	3	4
1610	1991	usa	2	3	4
1611	1998	chn	2	3	4
1612	1996	aus	2	3	4
1613	1989	ger	2	3	4
1614	1997	usa	2	3	4
1615	1996	hun	2	3	4
1616	1992	chn	2	3	4
1617	1989	hun	2	3	5
1618	1996	jpn	2	3	5
1619	1995	gbr	2	3	5
1620	1993	usa	2	3	5
1621	1989	gbr	2	3	5
1622	1997	can	2	3	5
1623	1991	usa	2	3	5
1624	1996	chn	2	3	5
1625	1989	hun	2	4	5
1626	1993	usa	2	4	5
1627	1997	can	2	4	5
1628	1989	gbr	2	4	5
1629	1993	cze	2	4	5
1630	1992	jpn	2	4	5
1631	1993	gbr	2	4	5

1632	1991	fra	2	4	5
1633	1990	nzl	1	1	1
1634	1990	ita	1	1	1
1635	1991	ita	1	1	1
1636	1990	rus	1	1	1
1637	1991	ind	1	1	1
1638	1990	pol	1	1	1
1639	1991	bra	1	1	1
1640	1990	gre	1	1	1
1641	1990	ita	1	2	1
1642	1991	ita	1	2	1
1643	1990	rus	1	2	1
1644	1990	ger	1	2	1
1645	1990	aus	1	2	1
1646	1990	ven	1	2	1
1647	1991	ind	1	2	1
1648	1990	ger	1	2	1
1649	1991	rus	1	3	1
1650	1990	gbr	1	3	1
1651	1990	ger	1	3	1
1652	1992	lux	1	3	1
1653	1990	ita	1	3	1
1654	1991	ind	1	3	1
1655	1990	esp	1	3	1
1656	1990	usa	1	3	1
1657	1991	rus	1	4	1
1658	1990	ita	1	4	1
1659	1991	pol	1	4	1
1660	1990	fra	1	4	1
1661	1990	rsa	1	4	1
1662	1990	jpn	1	4	1
1663	1990	rus	1	4	1
1664	1990	mex	1	4	1
1665	1990	rsa	1	5	1
1666	1991	pol	1	5	1
1667	1990	fra	1	5	1
1668	1990	rus	1	5	1
1669	1992	jpn	1	5	1
1670	1990	ita	1	5	1
1671	1992	usa	1	5	1
1672	1990	nzl	1	5	1
1673	1991	pol	1	6	1
1674	1990	rus	1	6	1
1675	1990	fra	1	6	1
1676	1990	rsa	1	6	1
1677	1992	jpn	1	6	1
1678	19900	nzl	1	6	1
1679	1990	rus	1	6	1
1680	1992	usa	1	6	1
1681	1991	aus	1	1	2
1682	1990	blr	1	1	2

1683	1990	rus	1	1	2
1684	1990	pol	1	1	2
1685	1990	arg	1	1	2
1686	1991	ita	1	1	2
1687	1990	fra	1	1	2
1688	1990	rsa	1	1	2
1689	1990	nzl	1	2	2
1690	1991	aus	1	2	2
1691	1990	gbr	1	2	2
1692	1991	rsa	1	2	2
1693	1990	nzl	1	2	2
1694	1991	can	1	2	2
1695	1990	rus	1	2	2
1696	1990	rus	1	2	2
1697	1990	nzl	1	3	2
1698	1991	can	1	3	2
1699	1991	pol	1	3	2
1700	1990	usa	1	3	2
1701	1990	rus	1	3	2
1702	1990	fra	1	3	2
1703	1992	jpn	1	3	2
1704	1990	rsa	1	3	2
1705	1990	gbr	1	1	3
1706	1990	srb	1	1	3
1707	1991	ita	1	1	3
1708	1990	ger	1	1	3
1709	1991	rus	1	1	3
1710	1992	ukr	1	1	3
1711	1990	jpn	1	1	3
1712	1990	pol	1	1	3
1713	1990	gbr	1	2	3
1714	1990	ger	1	2	3
1715	1990	srb	1	2	3
1716	1991	ita	1	2	3
1717	1992	ukr	1	2	3
1718	1990	usa	1	2	3
1719	1990	can	1	2	3
1720	1991	rus	1	2	3
1721	1990	rus	1	3	3
1722	1990	ger	1	3	3
1723	1990	gbr	1	3	3
1724	1990	usa	1	3	3
1725	1991	ger	1	3	3
1726	1991	rus	1	3	3
1727	1990	esp	1	3	3
1728	1991	aus	1	3	3
1729	1990	nzl	1	1	4
1730	1990	srb	1	1	4
1731	1990	blr	1	1	4
1732	1990	usa	1	1	4
1733	1990	gbr	1	1	4

1734	1991	bra	1	1	4
1735	1991	jpn	1	1	4
1736	1990	bra	1	1	4
1737	1990	nzl	1	2	4
1738	1990	srb	1	2	4
1739	1990	usa	1	2	4
1740	1990	gbr	1	2	4
1741	1991	bra	1	2	4
1742	1991	aus	1	2	4
1743	1991	jpn	1	2	4
1744	1993	jpn	1	2	4
1745	1990	jpn	1	3	4
1746	1990	ita	1	3	4
1747	1991	jpn	1	3	4
1748	1991	aus	1	3	4
1749	1990	cze	1	3	4
1750	1990	ita	1	3	4
1751	1991	bra	1	3	4
1752	1990	ukr	1	3	4
1753	1990	ger	1	3	5
1754	1990	usa	1	3	5
1755	1990	jpn	1	3	5
1756	1991	bra	1	3	5
1757	1990	can	1	3	5
1758	1990	jpn	1	3	5
1759	1990	ita	1	3	5
1760	1991	can	1	3	5
1761	1991	usa	1	4	5
1762	1990	ger	1	4	5
1763	1991	jpn	1	4	5
1764	1990	mex	1	4	5
1765	1992	pol	1	4	5
1766	1990	ita	1	4	5
1767	1992	chn	1	4	5
1768	1990	jpn	1	4	5
1769	1992	fra	2	1	1
1770	1991	can	2	1	1
1771	1991	swe	2	1	1
1772	1991	jpn	2	1	1
1773	1992	gre	2	1	1
1774	1991	chn	2	1	1
1775	1994	ger	2	1	1
1776	1992	pol	2	1	1
1777	1991	usa	2	2	1
1778	1994	ger	2	2	1
1779	1991	usa	2	2	1
1780	1991	swe	2	2	1
1781	1992	pol	2	2	1
1782	1992	esp	2	2	1
1783	1991	chn	2	2	1
1784	1991	ger	2	2	1

1785	1992	usa	2	3	1
1786	1991	usa	2	3	1
1787	1992	aus	2	3	1
1788	1991	jpn	2	3	1
1789	1992	esp	2	3	1
1790	1993	can	2	3	1
1791	1992	rus	2	3	1
1792	1992	slo	2	3	1
1793	1991	rus	2	4	1
1794	1992	fra	2	4	1
1795	1991	usa	2	4	1
1796	1991	gbr	2	4	1
1797	1991	can	2	4	1
1798	1992	usa	2	4	1
1799	1991	ven	2	4	1
1800	1991	jpn	2	4	1
1801	1991	rus	2	5	1
1802	1991	usa	2	5	1
1803	1991	ven	2	5	1
1804	1991	can	2	5	1
1805	1992	usa	2	5	1
1806	1991	can	2	5	1
1807	1992	fra	2	5	1
1808	1991	gre	2	5	1
1809	1991	rus	2	6	1
1810	1991	gre	2	6	1
1811	1991	ven	2	6	1
1812	1992	usa	2	6	1
1813	1992	fra	2	6	1
1814	1991	usa	2	6	1
1815	1991	ita	2	6	1
1816	1994	slo	2	6	1
1817	1991	aus	2	1	2
1818	1991	bra	2	1	2
1819	1993	usa	2	1	2
1820	1991	can	2	1	2
1821	1991	pol	2	1	2
1822	1994	jpn	2	1	2
1823	1991	can	2	1	2
1824	1991	gre	2	1	2
1825	1991	aus	2	2	2
1826	1993	usa	2	2	2
1827	1991	can	2	2	2
1828	1991	pol	2	2	2
1829	1991	can	2	2	2
1830	1994	jpn	2	2	2
1831	1994	usa	2	2	2
1832	1994	jpn	2	2	2
1833	1991	pol	2	3	2
1834	1993	usa	2	3	2
1835	1992	pol	2	3	2

1836	1991 can	2	3	2
1837	1993 jpn	2	3	2
1838	1994 usa	2	3	2
1839	1991 gbr	2	3	2
1840	1992 ita	2	3	2
1841	1991 usa	2	1	3
1842	1991 usa	2	1	3
1843	1993 can	2	1	3
1844	1991 chn	2	1	3
1845	1992 aus	2	1	3
1846	1992 rus	2	1	3
1847	1994 ken	2	1	3
1848	1991 swe	2	1	3
1849	1992 aus	2	2	3
1850	1991 jpn	2	2	3
1851	1992 rus	2	2	3
1852	1993 rus	2	2	3
1853	1991 chn	2	2	3
1854	1992 jpn	2	2	3
1855	1991 usa	2	2	3
1856	1993 can	2	2	3
1857	1993 rus	2	3	3
1858	1993 jpn	2	3	3
1859	1991 usa	2	3	3
1860	1991 jpn	2	3	3
1861	1992 rus	2	3	3
1862	1992 gbr	2	3	3
1863	1991 can	2	3	3
1864	1992 pol	2	3	3
1865	1993 ita	2	1	4
1866	1992 fra	2	1	4
1867	19991 aus	2	1	4
1868	1993 ger	2	1	4
1869	1992 jpn	2	1	4
1870	1992 gre	2	1	4
1871	1994 ger	2	1	4
1872	1993 usa	2	1	4
1873	1991 jpn	2	2	4
1874	1993 ita	2	2	4
1875	1992 jpn	2	2	4
1876	1991 usa	2	2	4
1877	1993 ger	2	2	4
1878	1991 swe	2	2	4
1879	1991 can	2	2	4
1880	1991 can	2	2	4
1881	1991 jpn	2	3	4
1882	1991 ger	2	3	4
1883	1992 jpn	2	3	4
1884	1991 usa	2	3	4
1885	1991 usa	2	3	4
1886	1992 pol	2	3	4

1887	1992	gre	2	3	4
1888	1991	esp	2	3	4
1889	1992	usa	2	3	5
1890	1992	aus	2	3	5
1891	1992	rus	2	3	5
1892	1992	ger	2	3	5
1893	1994	chn	2	3	5
1894	1991	fra	2	3	5
1895	1992	jpn	2	3	5
1896	1994	chn	2	3	5
1897	1992	usa	2	4	5
1898	1992	aus	2	4	5
1899	1991	can	2	4	5
1900	1992	usa	2	4	5
1901	1993	irl	2	4	5
1902	1992	pol	2	4	5
1903	1991	fra	2	4	5
1904	1992	slo	2	4	5
1905	1994	aus	1	1	1
1906	1993	esp	1	1	1
1907	1993	gre	1	1	1
1908	1994	ukr	1	1	1
1909	1993	fra	1	1	1
1910	1993	can	1	1	1
1911	1993	lit	1	1	1
1912	1994	south africa	1	1	1
1913	1994	aus	1	2	1
1914	1993	rus	1	2	1
1915	1993	pol	1	2	1
1916	1994	ita	1	2	1
1917	1993	usa	1	2	1
1918	1993	can	1	2	1
1919	1993	rus	1	2	1
1920	1993	latvia	1	2	1
1921	1993	can	1	3	1
1922	1993	pol	1	3	1
1923	1994	aus	1	3	1
1924	1994	jpn	1	3	1
1925	1995	ita	1	3	1
1926	1993	rus	1	3	1
1927	1993	usa	1	3	1
1928	1994	usa	1	3	1
1929	1994	jpn	1	4	1
1930	1993	can	1	4	1
1931	1994	usa	1	4	1
1932	1994	ita	1	4	1
1933	1995	ita	1	4	1
1934	1995	gb	1	4	1
1935	1995	usa	1	4	1
1936	1993	can	1	4	1
1937	1994	usa	1	5	1

1938	1994 ita	1	5	1
1939	1994 ita	1	5	1
1940	1994 aus	1	5	1
1941	1995 gb	1	5	1
1942	1993 can	1	5	1
1943	1994 esp	1	5	1
1944	1994 pol	1	5	1
1945	1994 usa	1	6	1
1946	1994 ita	1	6	1
1947	1994 ita	1	6	1
1948	1994 aus	1	6	1
1949	1993 can	1	6	1
1950	1994 can	1	6	1
1951	1994 esp	1	6	1
1952	1993 chn	1	6	1
1953	1993 ger	1	1	2
1954	1993 usa	1	1	2
1955	1994 ita	1	1	2
1956	1993 ita	1	1	2
1957	1993 pol	1	1	2
1958	1993 gre	1	1	2
1959	1993 aus	1	1	2
1960	1994 usa	1	1	2
1961	1993 usa	1	2	2
1962	1994 ita	1	2	2
1963	1994 jpn	1	2	2
1964	1994 usa	1	2	2
1965	1993 ger	1	2	2
1966	1993 aus	1	2	2
1967	1994 can	1	2	2
1968	1994 bra	1	2	2
1969	1993 usa	1	3	2
1970	1994 jpn	1	3	2
1971	1995 usa	1	3	2
1972	1994 ita	1	3	2
1973	1993 ger	1	3	2
1974	1994 can	1	3	2
1975	1993 aus	1	3	2
1976	1993 chn	1	3	2
1977	1993 gre	1	1	3
1978	1994 gb	1	1	3
1979	1994 jpn	1	1	3
1980	1994 rus	1	1	3
1981	1993 bra	1	1	3
1982	1993 ger	1	1	3
1983	1993 usa	1	1	3
1984	1993 kaz	1	1	3
1985	1994 gb	1	2	3
1986	1994 jpn	1	2	3
1987	1993 gre	1	2	3
1988	1993 ukr	1	2	3

1989	1993 usa	1	2	3
1990	1993 rus	1	2	3
1991	1993 ita	1	2	3
1992	1993 kaz	1	2	3
1993	1994 jpn	1	3	3
1994	1993 ukr	1	3	3
1995	1993 ukr	1	3	3
1996	1994 usa	1	3	3
1997	1994 rus	1	3	3
1998	1993 usa	1	3	3
1999	1993 ita	1	3	3
2000	1993 aus	1	3	3
2001	1993 usa	1	1	4
2002	1994 cro	1	1	4
2003	1994 ita	1	1	4
2004	1993 ukr	1	1	4
2005	1993 usa	1	1	4
2006	1993 fra	1	1	4
2007	1993 chn	1	1	4
2008	1994 south africa	1	1	4
2009	1993 usa	1	2	4
2010	1994 jpn	1	2	4
2011	1993 bra	1	2	4
2012	1993 usa	1	2	4
2013	1995 sing	1	2	4
2014	1993 ukr	1	2	4
2015	1993 belarus	1	2	4
2016	1994 rus	1	2	4
2017	1994 jpn	1	3	4
2018	1994 gre	1	3	4
2019	1993 can	1	3	4
2020	1993 jpn	1	3	4
2021	1994 rus	1	3	4
2022	1993 south africa	1	3	4
2023	1993 can	1	3	4
2024	1993 gb	1	3	4
2025	1994 jpn	1	3	5
2026	1994 gre	1	3	5
2027	1993 ukr	1	3	5
2028	1994 usa	1	3	5
2029	1993 usa	1	3	5
2030	1993 south africa	1	3	5
2031	1993 esp	1	3	5
2032	1995 rus	1	3	5
2033	1993 ukr	1	4	5
2034	1993 chn	1	4	5
2035	1994 jpn	1	4	5
2036	1993 usa	1	4	5
2037	1995 gb	1	4	5
2038	1994 usa	1	4	5
2039	1993 can	1	4	5

2040	1993 can	1	4	5
2041	1995 aus	2	1	1
2042	1995 usa	2	1	1
2043	1994 can	2	1	1
2044	1995 usa	2	1	1
2045	1995 ukr	2	1	1
2046	1995 fra	2	1	1
2047	1994 bra	2	1	1
2048	1994 jpn	2	1	1
2049	1995 usa	2	2	1
2050	1994 can	2	2	1
2051	1995 aus	2	2	1
2052	1996 usa	2	2	1
2053	1995 gb	2	2	1
2054	1994 jpn	2	2	1
2055	1996 aus	2	2	1
2056	1995 ger	2	2	1
2057	1994 can	2	3	1
2058	1994 usa	2	3	1
2059	1996 chn	2	3	1
2060	1994 jpn	2	3	1
2061	1995 usa	2	3	1
2062	1995 aus	2	3	1
2063	1994 slo	2	3	1
2064	1996 rus	2	3	1
2065	1994 can	2	4	1
2066	1995 aus	2	4	1
2067	1995 usa	2	4	1
2068	1994 jpn	2	4	1
2069	1994 usa	2	4	1
2070	1994 esp	2	4	1
2071	1995 aus	2	4	1
2072	1994 ukr	2	4	1
2073	1995 aus	2	5	1
2074	1995 usa	2	5	1
2075	1994 esp	2	5	1
2076	1994 can	2	5	1
2077	1994 usa	2	5	1
2078	1994 slo	2	5	1
2079	1995 pol	2	5	1
2080	1994 jpn	2	5	1
2081	1994 slo	2	6	1
2082	1994 usa	2	6	1
2083	1994 esp	2	6	1
2084	1996 south africa	2	6	1
2085	1996 esp	2	6	1
2086	1995 auus	2	6	1
2087	1995 usa	2	6	1
2088	1995 pol	2	6	1
2089	1994 ukr	2	1	2
2090	1994 gb	2	1	2

2091	1994	can	2	1	2
2092	1994	tur	2	1	2
2093	1994	usa	2	1	2
2094	1996	fra	2	1	2
2095	1994	fra	2	1	2
2096	1994	kaz	2	1	2
2097	1994	ukr	2	2	2
2098	1996	chn	2	2	2
2099	1994	gb	2	2	2
2100	1994	can	2	2	2
2101	1996	usa	2	2	2
2102	1994	can	2	2	2
2103	1994	usa	2	2	2
2104	1995	aus	2	2	2
2105	1994	ukr	2	3	2
2106	1994	jpn	2	3	2
2107	1994	gb	2	3	2
2108	1994	can	2	3	2
2109	1994	gb	2	3	2
2110	1994	usa	2	3	2
2111	1995	aus	2	3	2
2112	1996	chn	2	3	2
2113	1994	ita	2	1	3
2114	1994	usa	2	1	3
2115	1995	fra	2	1	3
2116	1996	jpn	2	1	3
2117	1994	ger	2	1	3
2118	1995	malaysia	2	1	3
2119	1994	usa	2	1	3
2120	1994	slo	2	1	3
2121	1994	ita	2	2	3
2122	1996	jpn	2	2	3
2123	1995	rus	2	2	3
2124	1994	esp	2	2	3
2125	1994	ger	2	2	3
2126	1995	rus	2	2	3
2127	1995	fra	2	2	3
2128	1994	slo	2	2	3
2129	1996	jpn	2	3	3
2130	1994	ita	2	3	3
2131	1995	rus	2	3	3
2132	1995	rus	2	3	3
2133	1994	esp	2	3	3
2134	1994	jpn	2	3	3
2135	1994	usa	2	3	3
2136	1995	aus	2	3	3
2137	1995	egy	2	1	4
2138	1994	usa	2	1	4
2139	1994	can	2	1	4
2140	1995	south africa	2	1	4
2141	1994	fra	2	1	4

2142	1995	rus	2	1	4
2143	1995	ukr	2	1	4
2144	1995	jpn	2	1	4
2145	1994	gb	2	2	4
2146	1995	jpn	2	2	4
2147	1995	ger	2	2	4
2148	1994	esp	2	2	4
2149	1994	usa	2	2	4
2150	1995	south africa	2	2	4
2151	1994	fra	2	2	4
2152	1996	can	2	2	4
2153	1994	esp	2	3	4
2154	1994	ita	2	3	4
2155	1994	jpn	2	3	4
2156	1994	gb	2	3	4
2157	1994	usa	2	3	4
2158	1994	usa	2	3	4
2159	1994	can	2	3	4
2160	1996	can	2	3	4
2161	1994	eso	2	3	5
2162	1996	jpn	2	3	5
2163	1995	can	2	3	5
2164	1994	aus	2	3	5
2165	1995	aus	2	3	5
2166	1994	usa	2	3	5
2167	1994	ita	2	3	5
2168	1995	rus	2	3	5
2169	1994	jpn	2	4	5
2170	1994	ita	2	4	5
2171	1994	esp	2	4	5
2172	1996	jpn	2	4	5
2173	1995	esp	2	4	5
2174	1994	usa	2	4	5
2175	1994	usa	2	4	5
2176	1994	chn	2	4	5
2177	1995	aus	1	1	1
2178	1996	rus	1	1	1
2179	1996	usa	1	1	1
2180	1996	bra	1	1	1
2181	1995	usa	1	1	1
2182	1996	pol	1	1	1
2183	1996	ukr	1	1	1
2184	1995	suriname	1	1	1
2185	1996	usa	1	2	1
2186	1995	aus	1	2	1
2187	1996	rus	1	2	1
2188	1995	pol	1	2	1
2189	1995	aus	1	2	1
2190	1995	south africa	1	2	1
2191	1996	ita	1	2	1
2192	1995	rus	1	2	1

2193	1996	aus	1	3	1
2194	1995	gb	1	3	1
2195	1995	ita	1	3	1
2196	1996	ita	1	3	1
2197	1995	rjus	1	3	1
2198	1996	usa	1	3	1
2199	1995	aus	1	3	1
2200	1996	bra	1	3	1
2201	1996	aus	1	4	1
2202	1995	gb	1	4	1
2203	1995	czech republic	1	4	1
2204	1995	ita	1	4	1
2205	1995	fra	1	4	1
2206	1996	ita	1	4	1
2207	1995	pol	1	4	1
2208	1996	can	1	4	1
2209	1996	aus	1	5	1
2210	1995	czech republic	1	5	1
2211	1995	pol	1	5	1
2212	1995	fra	1	5	1
2213	1996	egy	1	5	1
2214	1995	gb	1	5	1
2215	1996	can	1	5	1
2216	1996	pol	1	5	1
2217	1996	aus	1	6	1
2218	1995	czech republic	1	6	1
2219	1995	pol	1	6	1
2220	1996	ukr	1	6	1
2221	1995	fra	1	6	1
2222	1995	usa	1	6	1
2223	1996	pol	1	6	1
2224	1996	egy	1	6	1
2225	1995	rus	1	1	2
2226	1995	ger	1	1	2
2227	1995	gre	1	1	2
2228	1996	bra	1	1	2
2229	1996	ita	1	1	2
2230	1995	aus	1	1	2
2231	1995	lit	1	1	2
2232	1996	trinidad & tob	1	1	2
2233	1996	gre	1	2	2
2234	1995	lit	1	2	2
2235	1995	rus	1	2	2
2236	1996	trinidad & tob	1	2	2
2237	1995	jpn	1	2	2
2238	1995	ita	1	2	2
2239	1996	usa	1	2	2
2240	1996	bra	1	2	2
2241	1995	ita	1	3	2
2242	1995	jpn	1	3	2
2243	1996	usa	1	3	2

2244	1996	usa	1	3	2
2245	1995	lit	1	3	2
2246	1995	hun	1	3	2
2247	1996	south africa	1	3	2
2248	1995	swe	1	3	2
2249	1995	slo	1	1	3
2250	1995	jpn	1	1	3
2251	1995	rus	1	1	3
2252	1995	bra	1	1	3
2253	1995	uzbekistan	1	1	3
2254	1997	usa	1	1	3
2255	1996	pol	1	1	3
2256	1995	pol	1	1	3
2257	1995	rus	1	2	3
2258	1995	rus	1	2	3
2259	1995	jpn	1	2	3
2260	1995	kaz	1	2	3
2261	1996	ven	1	2	3
2262	1996	pol	1	2	3
2263	1995	bra	1	2	3
2264	1995	can	1	2	3
2265	1995	rus	1	3	3
2266	1996	hun	1	3	3
2267	1995	rus	1	3	3
2268	1997	jpn	1	3	3
2269	1996	usa	1	3	3
2270	1995	kaz	1	3	3
2271	1996	ven	1	3	3
2272	1995	usa	1	3	3
2273	1996	aus	1	1	4
2274	1996	trinidad & tob	1	1	4
2275	1996	jpn	1	1	4
2276	1995	ger	1	1	4
2277	1996	usa	1	1	4
2278	1995	usa	1	1	4
2279	1995	south africa	1	1	4
2280	1995	lit	1	1	4
2281	1996	jpn	1	2	4
2282	1995	bra	1	2	4
2283	1996	usa	1	2	4
2284	1995	usa	1	2	4
2285	1995	rus	1	2	4
2286	1995	jpn	1	2	4
2287	1995	south africa	1	2	4
2288	1996	aus	1	2	4
2289	1996	usa	1	3	4
2290	1995	jpn	1	3	4
2291	1995	rus	1	3	4
2292	1995	gb	1	3	4
2293	1996	bra	1	3	4
2294	1995	aus	1	3	4

2295	1995	aus	1	3	4
2296	1996	pol	1	3	4
2297	1996	usa	1	3	5
2298	1995	rus	1	3	5
2299	1995	jpn	1	3	5
2300	1996	usa	1	3	5
2301	1996	can	1	3	5
2302	1995	gb	1	3	5
2303	1995	hun	1	3	5
2304	1995	rus	1	3	5
2305	1996	usa	1	4	5
2306	1995	rus	1	4	5
2307	1995	jpn	1	4	5
2308	1995	rus	1	4	5
2309	1995	can	1	4	5
2310	1995	usa	1	4	5
2311	1995	gb	1	4	5
2312	1995	gb	1	4	5
2313	1997	lit	2	1	1
2314	1997	rus	2	1	1
2315	1997	hong kong	2	1	1
2316	1997	ita	2	1	1
2317	1996	usa	2	1	1
2318	1997	fra	2	1	1
2319	1998	aus	2	1	1
2320	1998	bra	2	1	1
2321	1997	hong kong	2	2	1
2322	1997	lit	2	2	1
2323	1998	aus	2	2	1
2324	1996	usa	2	2	1
2325	1997	rus	2	2	1
2326	1996	gb	2	2	1
2327	1996	usa	2	2	1
2328	1997	rus	2	2	1
2329	1996	ita	2	3	1
2330	1997	rus	2	3	1
2331	1996	usa	2	3	1
2332	1997	hong kong	2	3	1
2333	1997	aus	2	3	1
2334	1996	can	2	3	1
2335	1997	aus	2	3	1
2336	1996	ger	2	3	1
2337	1997	aus	2	4	1
2338	1997	aus	2	4	1
2339	1996	usa	2	4	1
2340	1997	usa	2	4	1
2341	1996	ita	2	4	1
2342	1998	mex	2	4	1
2343	1996	can	2	4	1
2344	1998	ita	2	4	1
2345	1997	aus	2	5	1

2346	1997	usa	2	5	1
2347	1998	ita	2	5	1
2348	1999	usa	2	5	1
2349	1996	jpn	2	5	1
2350	1996	aus	2	5	1
2351	1997	ita	2	5	1
2352	1997	mex	2	5	1
2353	1997	usa	2	6	1
2354	1998	ita	2	6	1
2355	1999	usa	2	6	1
2356	1997	ita	2	6	1
2357	1996	aus	2	6	1
2358	1996	por	2	6	1
2359	1997	slo	2	6	1
2360	1996	can	2	6	1
2361	1999	new zealand	2	1	2
2362	1998	rus	2	1	2
2363	1996	usa	2	1	2
2364	1997	usa	2	1	2
2365	1997	lit	2	1	2
2366	1996	gb	2	1	2
2367	1996	ukr	2	1	2
2368	1998	ger	2	1	2
2369	1998	rus	2	2	2
2370	1997	usa	2	2	2
2371	1996	gb	2	2	2
2372	1996	usa	2	2	2
2373	1996	gb	2	2	2
2374	1997	aus	2	2	2
2375	1996	aus	2	2	2
2376	1996	can	2	2	2
2377	1996	usa	2	3	2
2378	1997	usa	2	3	2
2379	1998	rus	2	3	2
2380	1996	ukr	2	3	2
2381	1997	can	2	3	2
2382	1997	aus	2	3	2
2383	1999	south africa	2	3	2
2384	1996	jpn	2	3	2
2385	1997	lit	2	1	3
2386	1998	ukr	2	1	3
2387	1996	gb	2	1	3
2388	1997	ita	2	1	3
2389	1997	aus	2	1	3
2390	1996	hun	2	1	3
2391	1996	sweden	2	1	3
2392	1997	hun	2	1	3
2393	1997	lit	2	2	3
2394	1996	gb	2	2	3
2395	1998	ukr	2	2	3
2396	1996	rus	2	2	3

2397	1998	ger	2	2	3
2398	1997	usa	2	2	3
2399	1996	gb	2	2	3
2400	1996	ger	2	2	3
2401	1998	ukr	2	3	3
2402	1997	ukr	2	3	3
2403	1997	ita	2	3	3
2404	1996	gb	2	3	3
2405	1996	south africa	2	3	3
2406	1997	aus	2	3	3
2407	1998	ger	2	3	3
2408	1996	usa	2	3	3
2409	1996	rus	2	1	4
2410	1999	aus	2	1	4
2411	1997	czech republic	2	1	4
2412	1997	aus	2	1	4
2413	1997	can	2	1	4
2414	1997	usa	2	1	4
2415	1996	hun	2	1	4
2416	1997	fra	2	1	4
2417	1996	rus	2	2	4
2418	1996	hun	2	2	4
2419	1997	aus	2	2	4
2420	1997	czech republic	2	2	4
2421	1997	usa	2	2	4
2422	1996	usa	2	2	4
2423	1996	jpn	2	2	4
2424	1997	ita	2	2	4
2425	1997	usa	2	3	4
2426	1996	hun	2	3	4
2427	1996	jpn	2	3	4
2428	1997	aus	2	3	4
2429	1997	usa	2	3	4
2430	1997	jpn	2	3	4
2431	1997	gb	2	3	4
2432	1999	can	2	3	4
2433	1997	lit	2	3	5
2434	1997	usa	2	3	5
2435	1997	can	2	3	5
2436	1997	usa	2	3	5
2437	1996	jpn	2	3	5
2438	1999	jpn	2	3	5
2439	1997	hun	2	3	5
2440	1997	south africa	2	3	5
2441	1997	usa	2	4	5
2442	1997	usa	2	4	5
2443	1997	can	2	4	5
2444	1996	jpn	2	4	5
2445	1996	rus	2	4	5
2446	1997	can	2	4	5
2447	1999	jpn	2	4	5

2448	1997	south africa	2	4	5

T2J2006	T3J2006	TiempoJ2006	PuestoJ2006	T1A1	T2A1	T3A1
25	55		1			
25	61		2			
25	71		3			
26	4		4			
26	15		5			
26	22		6			
26	31		7			
26	42		8			
55	59		1			
55	96		2			
56	47		3			
56	60		4			
57	35		5			
57	41		6			
57	62		7			
57	78		8			
0	44		1			
1	26		2			
2	4		3			
3	26		4			
3	52		5			
3	99		6			
5	23		7			
7	2		8			
14	29		1			
14	45		2			
14	49		3			
15	34		4			
20	26		5			
20	79		6			
21	63		7			
21	99		8			
38	91		1			
40	69		2			
40	81		3			
41	16		4			
45	14		5			
49	15		6			
49	80		7			
53	40		8			
35	32		1			
40	99		2			
41	36		3			
46	79		4			
51	79		5			
56	33		6			
4	10		7			
5	36		8			
29	49		1			
29	58		2			

29	61	3
29	63	4
29	95	5
29	98	6
30	4	7
30	31	8
2	41	1
2	83	2
3	10	3
3	65	4
3	70	5
3	94	6
4	48	7
4	54	8
15	27	1
16	49	2
17	1	3
17	79	4
17	99	5
18	39	6
18	75	7
19	42	8
32	21	1
32	63	2
32	65	3
32	78	4
32	85	5
32	97	6
33	5	7
33	49	8
9	21	1
9	35	2
9	94	3
10	43	4
10	71	5
11	52	6
12	24	7
12	56	8
26	58	1
28	41	2
28	57	3
30	33	4
30	63	5
32	26	6
33	26	7
37	25	8
27	38	1
27	45	2
27	48	3
27	49	4
27	68	5

27	71	6
27	75	7
27	84	8
59	57	1
0	25	2
0	31	3
0	61	4
1	24	5
1	82	6
1	86	7
2	37	8
12	34	1
13	52	2
13	92	3
14	13	4
14	54	5
15	20	6
16	86	7
18	6	8
14	45	1
15	29	2
18	13	3
19	62	4
19	67	5
19	70	6
19	95	7
20	26	8
47	38	1
50	27	2
51	86	3
51	91	4
52	3	5
54	25	6
58	0	7
58	99	8
22	74	1
22	92	2
23	19	3
23	55	4
23	60	5
23	65	6
23	73	7
23	73	8
50	32	1
50	84	2
51	24	3
51	49	4
51	58	5
51	80	6
51	87	7
52	6	8

51	97	1
52	20	2
52	23	3
52	27	4
52	61	5
53	52	6
53	76	7
53	87	8
54	99	1
56	49	2
56	68	3
57	2	4
57	32	5
57	72	6
59	11	7
59	68	8
4	84	1
6	92	2
9	67	3
12	10	4
13	51	5
17	60	6
18	41	7
18	65	8
28	42	1
32	83	2
33	97	3
49	7	4
51	67	5
54	96	6
57	89	7
58	94	8
26	26	1
26	52	2
26	57	3
26	78	4
26	99	5
27	1	6
27	3	7
27	42	8
55	74	1
56	43	2
57	42	3
57	45	4
57	56	5
57	70	6
58	8	7
58	24	8
0	68	1
0	84	2
1	66	3

3	41	4
3	66	5
4	5	6
6	67	7
7	30	8
28	43	1
29	5	2
29	13	3
29	21	4
29	41	5
29	91	6
29	97	7
30	13	8
2	31	1
2	65	2
3	46	3
3	96	4
4	40	5
4	92	6
5	4	7
5	37	8
15	44	1
15	56	2
16	57	3
16	99	4
21	65	5
21	84	6
22	3	7
22	33	8
24	56	1
24	91	2
24	94	3
25	7	4
25	8	5
25	21	6
25	55	7
25	76	8
54	31	1
54	40	2
54	63	3
54	78	4
54	89	5
55	20	6
56	46	7
56	90	8
1	64	1
2	13	2
4	41	3
4	50	4
5	21	5
5	28	6

6	34		7			
6	42		8			
3	62		1			
4	69		2			
4	70		3			
4	71		4			
4	93		5			
6	29		6			
7	16		7			
7	70		8			
21	33		1			
23	42		2			
24	39		3			
24	56		4			
25	74		5			
28	82		6			
30	74		7			
31	22		8			
			1		21	88
			2		21	94
			3		21	97
			4		22	0
			5		22	5
			6		22	12
			7		22	16
			8		22	28
			1		48	43
			2		48	43
			3		48	47
			4		48	51
			5		48	52
			6		48	63
			7		48	72
			8		48	81
			1	1	43	86
			2	1	46	28
			3	1	46	73
			4	1	47	12
			5	1	47	18
			6	1	47	53
			7	1	48	9
			8	1	49	13
			1	3	44	30
			2	3	45	12
			3	3	45	43
			4	3	45	47
			5	3	46	36
			6	3	48	1
			7	3	48	26
			8	3	48	49
			1	7	46	95

2	7	47	91
3	7	48	67
4	7	49	98
5	7	52	4
6	7	53	43
7	7	55	39
8	7	56	56
1	14	45	94
2	14	47	29
3	14	51	21
4	14	52	98
5	14	56	22
6	14	59	11
7	14	59	59
8	15	7	76
1		24	98
2		25	20
3		25	23
4		25	29
5		25	32
6		25	52
7		25	56
8		25	61
1		52	98
2		53	50
3		53	61
4		53	69
5		53	78
6		54	59
7		54	65
8		55	4
1	1	54	32
2	1	54	80
3	1	56	2
4	1	57	14
5	1	57	31
6	1	58	88
7	1	59	14
8	1	59	41
1		27	66
2		27	69
3		27	88
4		28	9
5		28	10
6		28	19
7		28	24
8		28	27
1		59	80
2		59	96
3	1	0	58
4	1	0	60

5	1	0	83
6	1	1	17
7	1	1	24
8	1	1	67
1	2	9	80
2	2	10	99
3	2	11	3
4	2	11	38
5	2	11	50
6	2	11	62
7	2	12	1
8	2	12	16
1		23	18
2		23	47
3		23	56
4		23	57
5		23	61
6		23	70
7		23	86
8		24	14
1		50	77
2		50	82
3		51	82
4		52	3
5		52	23
6		52	53
7		52	54
8		52	70
1	1	52	9
2	1	55	13
3	1	55	22
4	1	55	35
5	1	55	81
6	1	55	87
7	1	56	48
8	1	58	15
1	1	54	98
2	1	56	19
3	1	56	92
4	1	58	98
5	1	59	46
6	1	59	57
7	1	59	84
8	2	0	73
1	4	6	22
2	4	9	74
3	4	9	88
4	4	11	68
5	4	14	76
6	4	15	75
7	4	16	83

8	4	17	32
1		24	53
2		24	62
3		24	70
4		24	79
5		24	83
6		24	96
7		25	2
8		25	31
1		53	40
2		53	70
3		53	74
4		53	87
5		54	10
6		54	21
7		54	67
8		54	77
1	1	55	52
2	1	55	68
3	1	56	97
4	1	57	9
5	1	57	90
6	1	58	30
7	1	59	28
8	2	1	53
1	4	2	61
2	4	4	23
3	4	5	19
4	4	5	65
5	4	5	79
6	4	6	99
7	4	7	42
8	4	7	64
1	8	18	52
2	8	18	80
3	8	26	41
4	8	27	59
5	8	28	23
6	8	31	73
7	8	32	60
8	8	34	96
1	15	53	5
2	15	55	38
3	15	58	55
4	16	5	83
5	16	12	84
6	16	20	82
7	16	27	13
8	16	42	17
1		28	16
2		28	46

3		28	50
4		28	54
5		28	64
6		28	70
7		28	86
8		28	87
1		59	44
2		59	87
3	1	0	40
4	1	0	52
5	1	0	63
6	1	0	79
7	1	1	38
8	1	2	68
1	2	7	16
2	2	7	54
3	2	8	54
4	2	9	59
5	2	10	57
6	2	10	66
7	2	11	9
8	2	11	41
1		30	63
2		30	70
3		31	5
4		31	14
5		31	35
6		31	79
7		31	82
8		31	86
1	1	5	72
2	1	6	34
3	1	7	27
4	1	7	38
5	1	8	5
6	1	8	55
7	1	8	72
8	1	8	96
1	2	21	84
2	2	25	94
3	2	25	94
4	2	26	19
5	2	27	55
6	2	28	13
7	2	28	25
8	2	28	67
1		25	91
2		26	5
3		26	11
4		26	32
5		26	41

6		26	77
7		26	80
8		27	6
1		57	15
2		57	24
3		57	34
4		58	30
5		58	34
6		58	73
7		58	76
8		59	22
1	2	6	39
2	2	6	71
3	2	6	90
4	2	7	22
5	2	7	73
6	2	9	43
7	2	9	66
8	2	13	61
1	2	10	13
2	2	10	76
3	2	11	42
4	2	13	73
5	2	14	5
6	2	14	89
7	2	15	26
8	2	15	28
1	4	32	89
2	4	40	14
3	4	41	19
4	4	41	53
5	4	41	87
6	4	44	49
7	4	45	61
8	4	46	97

2
3
4
5
6
7
8
1
2
3
4
5
6
7
8
1
2
3
4
5
6
7
8
1
2
3
4
5
6
7
8
1
2
3
4
5
6
7
8
1
2
3
4
5
6
7
8
1
2
3
4

5
6
7
8
1
2
3
4
5
6
7
8
1
2
3
4
5
6
7
8
1
2
3
4
5
6
7
8
1
2
3
4
5
6
7
8
1
2
3
4
5
6
7
8
1
2
3
4
5
6
7

8
1
2
3
4
5
6
7
8
1
2
3
4
5
6
7
8
1
2
3
4
5
6
7
8
1
2
3
4
5
6
7
8
1
2
3
4
5
6
7
8
1
2
3
4
5
6
7
8
1
2

3
4
5
6
7
8
1
2
3
4
5
6
7
8
1
2
3
4
5
6
7
8
1
2
3
4
5
6
7
8
1
2
3
4
5
6
7
8
1
2
3
4
5
6
7
8
1
2
3
4
5

6			
7			
8			
1			
2			
3			
4			
5			
6			
7			
8			
1			
2			
3			
4			
5			
6			
7			
8			
1			
2			
3			
4			
5			
6			
7			
8			
1			
2			
3			
4			
5			
6			
7			
8			
	23	73	1
	23	88	2
	23	99	3
	23	99	4
	24	11	5
	24	19	6
	24	23	7
	24	48	8
	52	7	1
	52	87	2
	52	93	3
	53	12	4
	53	30	5
	53	37	6
	53	70	7
	53	92	8

1	52	98	1
1	54	96	2
1	55	64	3
1	55	88	4
1	56	28	5
1	56	47	6
1	56	70	7
1	57	50	8
3	59	15	1
4	0	60	2
4	0	79	3
4	2	51	4
4	3	29	5
4	3	41	6
4	4	54	7
4	7	62	8
8	15	92	1
8	16	66	2
8	17	21	3
8	17	90	4
8	20	44	5
8	25	97	6
8	26	35	7
8	29	61	8
15	44	93	1
15	46	30	2
15	55	63	3
15	57	57	4
16	0	25	5
16	9	22	6
16	9	66	7
16	16	10	8
	27	6	1
	27	23	2
	27	28	3
	27	31	4
	27	62	5
	27	73	6
	27	83	7
	27	88	8
	58	12	1
	58	18	2
	58	88	3
	59	14	4
	59	28	5
	59	42	6
	59	71	7
	59	74	8
2	4	81	1
2	4	94	2
2	6	39	3

2	6	66	4
2	7	98	5
2	8	4	6
2	8	13	7
2	8	89	8
	30	9	1
	30	11	2
	30	16	3
	30	46	4
	30	53	5
	30	65	6
	30	67	7
	30	91	8
1	4	93	1
1	5	41	2
1	5	75	3
1	5	86	4
1	6	37	5
1	6	38	6
1	6	75	7
1	7	1	8
2	21	62	1
2	21	84	2
2	21	97	3
2	22	15	4
2	23	3	5
2	23	12	6
2	23	36	7
2	23	62	8
	25	48	1
	25	57	2
	25	58	3
	25	59	4
	25	63	5
	25	66	6
	25	92	7
	26	12	8
	56	6	1
	56	23	2
	56	86	3
	56	89	4
	56	94	5
	56	94	6
	56	96	7
	57	79	8
2	3	41	1
2	3	90	2
2	4	28	3
2	4	41	4
2	4	50	5
2	5	48	6

2	5	95	7
2	6	11	8
2	6	15	1
2	7	3	2
2	7	46	3
2	8	94	4
2	9	73	5
2	9	91	6
2	9	98	7
2	10	85	8
4	30	31	1
4	32	12	2
4	32	29	3
4	32	72	4
4	34	90	5
4	35	33	6
4	37	85	7
4	38	15	8
	21	8	1
	21	21	2
	21	25	3
	21	38	4
	21	47	5
	21	49	6
	21	51	7
	21	53	8
	46	91	1
	47	12	2
	47	25	3
	47	27	4
	47	33	5
	47	37	6
	47	94	7
	48	1	8
1	42	0	1
1	43	22	2
1	43	90	3
1	45	24	4
1	45	46	5
1	45	67	6
1	46	5	7
1	46	33	8
3	40	7	1
3	41	11	2
3	41	35	3
3	43	20	4
3	44	40	5
3	46	30	6
3	46	60	7
3	47	2	8
7	32	12	1

7	35	277	2
7	41	92	3
7	43	84	4
7	44	32	5
7	48	44	6
7	49	46	7
7	49	46	8
14	37	28	1
14	41	38	2
14	46	84	3
14	48	28	4
14	54	23	5
14	57	3	6
15	1	92	7
15	19	38	8
	24	4	1
	24	24	2
	24	34	3
	24	57	4
	24	61	5
	24	63	6
	24	87	7
	24	15	8
	52	26	1
	52	54	2
	52	64	3
	52	73	4
	52	73	5
	52	87	6
	53	14	7
	53	43	8
1	51	92	1
1	52	51	2
1	53	82	3
1	54	75	4
1	54	92	5
1	55	36	6
1	55	60	7
1	56	63	8
	26	67	1
	26	76	2
	26	86	3
	26	95	4
	26	95	5
	27	10	6
	27	31	7
	27	31	8
	58	58	1
	58	64	2
	58	95	3
	58	98	4

	59	23	5
	59	27	6
	59	33	7
	59	54	8
2	7	64	1
2	7	65	2
2	7	80	3
2	7	80	4
2	8	23	5
2	8	86	6
2	9	35	7
2	10	26	8
	22	67	1
	22	73	2
	22	88	3
	22	93	4
	23	0	5
	23	4	6
	23	7	7
	23	10	8
	49	82	1
	49	95	2
	50	41	3
	50	79	4
	50	85	5
	51	7	6
	51	42	7
	51	74	8
1	51	51	1
1	53	23	2
1	53	32	3
1	54	27	4
1	54	45	5
1	55	8	6
1	55	43	7
1	55	43	8
1	54	10	1
1	55	24	2
1	55	36	3
1	55	55	4
1	56	69	5
1	57	93	6
1	58	2	7
1	59	32	8
4	7	1	1
4	7	31	2
4	7	37	3
4	8	86	4
4	10	40	5
4	12	33	6
4	13	63	7

	4	13	63	8

	23	55	6
	23	60	7
	23	64	8
	50	71	1
	51	15	2
	51	26	3
	51	59	4
	51	75	5
	51	86	6
	52	21	7
	52	36	8
1	53	34	1
1	54	1	2
1	54	67	3
1	55	0	4
1	55	7	5
1	55	39	6
1	55	48	7
1	55	53	8
1	54	0	1
1	54	16	2
1	57	69	3
1	57	79	4
1	58	14	5
1	59	0	6
1	59	26	7
1	59	52	8
4	7	13	1
4	11	17	2
4	11	98	3
4	13	62	4
4	14	62	5
4	15	67	6
4	15	89	7
4	19	85	8
	24	14	1
	24	27	2
	24	49	3
	24	60	4
	24	65	5
	24	67	6
	24	79	7
	24	87	8
	53	45	1
	53	45	2
	53	66	3
	53	72	4
	53	72	5
	53	81	6
	54	19	7
	54	22	8

1	55	58	1
1	56	4	2
1	56	1	3
1	56	41	4
1	56	60	5
1	56	98	6
1	57	63	7
1	58	26	8
4	1	97	1
4	4	1	2
4	4	6	3
4	4	62	4
4	4	68	5
4	6	11	6
4	8	22	7
4	9	66	8
8	17	51	1
8	18	20	2
8	23	36	3
8	24	5	4
8	24	79	5
8	29	20	6
8	30	45	7
8	32	72	8
15	49	59	1
15	55	60	2
15	58	2	3
16	5	11	4
16	5	98	5
16	6	2	6
16	9	71	7
16	12	42	8
	27	79	1
	27	93	2
	28	1	3
	28	6	4
	28	7	5
	28	9	6
	28	9	7
	28	42	8
	59	5	1
	59	6	2
	59	15	3
	59	21	4
	59	35	5
	59	53	6
	59	89	7
1	0	20	8
2	5	10	1
2	6	6	2
2	7	78	3

2	7	82	4
2	8	16	5
2	8	69	6
2	8	76	7
2	9	7	8
	30	19	1
	30	49	2
	30	58	3
	30	74	4
	30	89	5
	31	1	6
	31	33	7
	31	45	8
1	5	5	1
1	6	25	2
1	6	52	3
1	6	56	4
1	7	8	5
1	7	28	6
1	7	64	7
1	7	97	8
2	21	47	1
2	22	22	2
2	24	81	3
2	25	9	4
2	25	36	5
2	25	93	6
2	26	56	7
2	27	0	8
	25	71	1
	25	76	2
	25	86	3
	25	87	4
	25	87	5
	26	2	6
	26	6	7
	26	21	8
	56	87	1
	56	94	2
	57	6	3
	57	38	4
	57	55	5
	57	57	6
	57	95	7
	57	96	8
2	5	55	1
2	5	59	2
2	5	90	3
2	5	91	4
2	6	8	5
2	6	35	6

2	6	64	7		
2	6	64	8		
2	8	90	1		
2	9	0	2		
2	9	12	3		
2	9	65	4		
2	10	40	5		
2	11	24	6		
2	11	36	7		
2	16	18	8		
4	31	78	1		
4	34	22	2		
4	34	23	3		
4	34	94	4		
4	35	15	5		
4	35	78	6		
4	38	4	7		
4	38	80	8		
			1		21
			2		21
			3		21
			4		21
			5		21
			6		21
			7		21
			8		21
			1		47
			2		47
			3		47
			4		47
			5		48
			6		48
			7		48
			8		48
			1	1	44
			2	1	45
			3	1	45
			4	1	45
			5	1	45
			6	1	46
			7	1	46
			8	1	47
			1	3	41
			2	3	44
			3	3	44
			4	3	45
			5	3	47
			6	3	48
			7	3	48
			8	3	48
			1	7	41

2	7	43
3	7	43
4	7	44
5	7	47
6	7	50
7	7	52
8	7	52
1	14	41
2	14	42
3	14	45
4	14	47
5	14	53
6	15	0
7	15	3
8	15	5
1		24
2		24
3		24
4		24
5		24
6		24
7		25
8		25
1		52
2		53
3		53
4		53
5		53
6		53
7		53
8		54
1	1	53
2	1	54
3	1	54
4	1	55
5	1	55
6	1	55
7	1	57
8	1	58
1		26
2		26
3		27
4		27
5		27
6		27
7		27
8		27
1		58
2		58
3		59
4		59

5		59
6		59
7	1	0
8	1	0
1	2	7
2	2	8
3	2	8
4	2	9
5	2	9
6	2	9
7	2	9
8	2	10
1		23
2		23
3		23
4		23
5		23
6		23
7		23
8		23
1		51
2		51
3		51
4		51
5		51
6		51
7		51
8		51
1	1	54
2	1	55
3	1	55
4	1	55
5	1	55
6	1	56
7	1	56
8	1	56
1	1	54
2	1	56
3	1	56
4	1	56
5	1	57
6	1	57
7	1	58
8	1	59
1	4	8
2	4	9
3	4	9
4	4	10
5	4	10
6	4	13
7	4	13

8	4	17
1		24
2		24
3		24
4		24
5		24
6		24
7		24
8		24
1		52
2		52
3		53
4		53
5		53
6		53
7		54
8		54
1	1	54
2	1	55
3	1	55
4	1	56
5	1	57
6	1	57
7	1	57
8	1	57
1	3	59
2	3	2
3	3	3
4	3	4
5	3	5
6	3	7
7	3	7
8	3	8
1	8	13
2	8	16
3	8	18
4	8	21
5	8	21
6	8	27
7	8	29
8	8	37
1	15	36
2	15	38
3	15	44
4	15	58
5	16	0
6	16	1
7	16	6
8	16	9
1		27
2		27

3		27
4		27
5		27
6		27
7		27
8		27
1	1	4
2	1	5
3	1	5
4	1	5
5	1	6
6	1	6
7	1	7
8	1	7
1	2	4
2	2	6
3	2	6
4	2	8
5	2	8
6	2	9
7	2	10
8	2	11
1		29
2		29
3		29
4		29
5		30
6		30
7		31
8	DSQ	
1		58
2		59
3		59
4		59
5		59
6		59
7	1	0
8	1	0
1	2	19
2	2	20
3	2	22
4	2	22
5	2	23
6	2	23
7	2	24
8	2	25
1		25
2		25
3		25
4		25
5		25

6		25
7		26
8		26
1		56
2		56
3		57
4		57
5		57
6		58
7		58
8		58
1	2	4
2	2	4
3	2	5
4	2	6
5	2	6
6	2	6
7	2	7
8	2	8
1	2	7
2	2	9
3	2	9
4	2	10
5	2	10
6	2	10
7	2	11
8	2	12
1	4	30
2	4	31
3	4	31
4	4	32
5	4	34
6	4	34
7	4	38
8	4	39

53	3
83	4
93	5
96	6
5	7
33	8
42	1
2	2
52	3
93	4
74	5
81	6
8	7
41	8
76	1
66	2
80	3
72	4
98	5
8	6
46	7
30	8
	1
	2
	3
	4
	5
	6
	7
	8
42	1
6	2
23	3
45	4
61	5
84	6
16	7
29	8
41	1
8	2
37	3
96	4
1	5
55	6
1	7
21	8
24	1
42	2
53	3
70	4
83	5

96	6		
17	7		
46	8		
53	1		
97	2		
24	3		
27	4		
97	5		
11	6		
13	7		
30	8		
59	1		
78	2		
59	3		
9	4		
58	5		
65	6		
73	7		
40	8		
92	1		
39	2		
45	3		
48	4		
73	5		
95	6		
32	7		
3	8		
41	1		
21	2		
69	3		
70	4		
16	5		
50	6		
51	7		
21	8		
		21	19
		21	52
		21	55
		21	56
		21	86
		21	86
		21	98
		22	4
		47	84
		47	95
		48	12
		48	19
		48	27
		48	28
		48	31
		48	31

	1	45	14
	1	45	20
	1	45	38
	1	45	83
	1	45	91
	1	46	53
	1	46	88
	1	47	26
	3	42	58
	3	43	75
	3	44	59
	3	44	81
	3	46	29
	3	46	81
	3	48	52
	3	51	94
	7	39	96
	7	40	81
	7	44	2
	7	44	51
	7	45	66
	7	45	90
	7	49	86
	7	55	30
	14	39	67
	14	41	20
	14	51	8
	14	53	66
	14	58	62
	14	6	81
	15	9	77
DNS			
		24	23
		24	61
		24	69
		24	70
		24	73
		24	84
		24	88
		24	95
		52	40
		52	48
		52	66
		52	89
		53	2
		53	10
		53	23
		53	37
	1	53	58
	1	54	55
	1	54	60

1	54	81
1	55	0
1	55	20
1	56	26
1	56	79
	26	51
	26	66
	26	86
	26	87
	27	23
	27	23
	27	36
	27	45
	58	52
	58	59
	59	9
	59	42
	59	44
	59	56
	59	84
1	0	16
2	7	76
2	8	5
2	8	10
2	8	52
2	9	12
2	9	58
2	9	96
2	10	2
	22	97
	23	9
	23	15
	23	15
	23	18
	23	21
	23	25
	23	39
	50	56
	50	87
	50	96
	51	6
	51	24
	51	28
	51	46
	51	66
1	53	48
1	53	68
1	54	10
1	54	24
1	54	66
1	55	16

1	55	39
1	56	17
1	55	81
1	56	65
1	56	81
1	57	59
1	57	96
1	58	14
1	58	52
1	59	6
4	8	50
4	9	90
4	10	5
4	11	71
4	12	8
4	13	77
4	13	81
4	16	74
	24	12
	24	22
	24	31
	24	36
	24	39
	24	44
	24	51
	24	57
	52	52
	52	70
	52	82
	53	17
	53	58
	53	93
	54	0
	54	76
1	55	16
1	55	32
1	55	49
1	56	16
1	56	19
1	56	27
1	56	41
1	56	79
3	59	13
4	3	2
4	3	34
4	3	74
4	4	38
4	6	50
4	7	30
4	8	22
8	7	39

8	17	65
8	18	15
8	18	41
8	21	36
8	22	93
8	23	67
8	24	12
15	25	48
15	40	14
15	47	9
15	49	0
15	52	17
16	3	74
16	6	55
16	9	57
	27	11
	27	26
	27	58
	27	66
	27	73
	27	92
	27	99
	28	17
	58	26
	58	75
	58	86
	59	2
	59	40
	59	66
	59	78
	59	99
2	5	81
2	6	34
2	6	84
2	7	64
2	8	49
2	8	51
2	8	66
2	9	53
	30	5
	30	11
	30	13
	30	14
	30	20
	30	74
	31	12
	31	14
1	5	66
1	6	36
1	6	42
1	6	43

1	6	55
1	7	10
1	7	17
1	7	60
2	21	15
2	22	44
2	22	76
2	22	76
2	22	76
2	23	19
2	23	59
2	23	61
	24	96
	25	34
	25	37
	25	64
	25	78
	25	85
	25	93
	26	20
	55	64
	57	5
	57	48
	57	67
	57	69
	57	85
	57	94
	58	22
2	5	56
2	6	40
2	6	51
2	6	78
2	6	78
2	6	95
2	7	76
2	10	20
2	6	12
2	8	45
2	8	77
2	8	99
2	10	19
2	10	32
2	10	41
2	14	1
4	30	39
4	31	71
4	32	52
4	34	79
4	36	73
4	37	19
4	38	75

4	40	98

PositionA5	T1J2008	T2J2008	T3J2008	TimeJ2008	PositionJ2008	T1J2011

7
8
1
2
3
4
5
6
7
8
1
2
3
4
5
6
7
8
1
2
3
4
5
6
7
8
1
2
3
4
5
6
7
8
1
2
3
4
5
6
7
8
1
2
3
4
5
6
7
8
1

2
3
4
5
6
7
8
1
2
3
4
5
6
7
8
1
2
3
4
5
6
7
8
1
2
3
4
5
6
7
8
1
2
3
4
5
6
7
8
1
2
3
4
5
6
7
8
1
2
3
4

5
6
7
8
1
2
3
4
5
6
7
8
1
2
3
4
5
6
7
8
1
2
3
4
5
6
7
8
1
2
3
4
5
6
7
8
1
2
3
4
5
6
7
8
1
2
3
4
5
6
7

8				
		22	37	1
		22	63	2
		22	70	3
		22	87	4
		22	95	5
		23	25	6
		23	27	7
		23	30	8
		50	6	1
		50	11	2
		50	33	3
		50	34	4
		50	57	5
		50	62	6
		50	70	7
		50	85	8
	1	47	63	1
	1	49	44	2
	1	49	72	3
	1	50	30	4
	1	50	32	5
	1	50	35	6
	1	51	40	7
	1	51	78	8
	3	51	81	1
	3	53	19	2
	3	53	66	3
	3	55	45	4
	3	55	90	5
	3	59	3	6
	3	59	70	7
	3	59	99	8
	8	1	77	1
	8	2	2	2
	8	4	71	3
	8	4	93	4
	8	10	55	5
	8	10	62	6
	8	13	6	7
	8	13	86	8
	15	25	1	1
	15	26	56	2
	15	31	7	3
	15	36	79	4
	15	36	81	5
	15	38	19	6
	15	38	61	7
	15	39	84	8
		25	60	1
		25	93	2

	26	17	3
	26	30	4
	26	39	5
	26	50	6
	26	53	7
	26	58	8
	55	32	1
	55	77	2
	56	4	3
	56	19	4
	56	26	5
	56	30	6
	56	38	7
	53	46	8
1	59	67	1
2	0	53	2
2	0	72	3
2	1	18	4
2	1	56	5
2	1	71	6
2	2	32	7
2	2	85	8
	28	37	1
	28	38	2
	28	40	3
	28	71	4
	28	94	5
	28	98	6
	29	7	7
	29	49	8
1	2	19	1
1	2	56	2
1	2	57	3
1	2	64	4
1	2	86	5
1	3	3	6
1	3	3	7
1	3	58	8
2	14	78	1
2	15	27	2
2	15	67	3
2	16	8	4
2	17	42	5
2	17	44	6
2	19	53	7
2	10	22	8
	23	61	1
	23	97	2
	24	26	3
	24	38	4
	24	45	5

	24	76	6
	24	88	7
	24	96	8
	52	52	1
	52	62	2
	52	87	3
	53	68	4
	54	26	5
	54	36	6
	54	41	7
	54	45	8
1	59	3	1
1	59	13	2
1	59	32	3
2	0	71	4
2	1	54	5
2	1	78	6
2	1	79	7
2	3	40	8
2	2	28	1
2	2	51	2
2	2	99	3
2	3	58	4
2	4	4	5
2	4	37	6
2	4	42	7
2	6	19	8
4	21	58	1
4	22	10	2
4	29	93	3
4	31	22	4
4	31	53	5
4	31	93	6
4	32	43	7
DQ			8
	25	88	1
	25	95	2
	26	1	3
	26	18	4
	26	21	5
	26	22	6
	26	38	7
	26	56	8
	55	59	1
	56	8	2
	56	32	3
	56	46	4
	56	69	5
	56	96	6
	57	4	7
	57	12	8

1	59	78	1
2	0	41	2
2	1	18	3
2	1	30	4
2	1	79	5
2	2	49	6
2	3	5	7
2	3	12	8
4	6	30	1
4	12	44	2
4	13	0	3
4	13	25	4
4	13	64	5
4	14	91	6
4	17	25	7
4	18	12	8
8	32	75	1
8	37	8	2
8	37	82	3
8	39	12	4
8	42	67	5
8	43	33	6
8	43	86	7
8	45	64	8
16	28	77	1
16	33	68	2
16	36	33	3
16	37	98	4
16	42	45	5
16	48	79	6
16	53	95	7
16	55	78	8
	28	83	1
	28	44	2
	28	45	3
	28	61	4
	28	67	5
	28	71	6
	28	91	7
	28	3	8
1	2	2	1
1	2	37	2
1	2	96	3
1	3	10	4
1	3	19	5
1	3	23	6
1	3	29	7
1	3	54	8
2	12	56	1
2	13	53	2
2	13	83	3

2	14	14	4
2	14	69	5
2	14	97	6
2	15	76	7
2	15	84	8
	31	81	1
	31	94	2
	32	2	3
	32	15	4
	32	25	5
	32	30	6
	32	49	7
	32	79	8
1	8	72	1
1	8	99	2
1	9	6	3
1	9	15	4
1	9	37	5
1	9	49	6
1	9	51	7
1	10	11	8
2	25	19	1
2	25	39	2
2	26	41	3
2	30	2	4
2	30	15	5
2	31	75	6
2	32	58	7
2	32	93	8
	26	77	1
	27	9	2
	27	13	3
	27	32	4
	27	47	5
	27	50	6
	27	51	7
	27	55	8
	59	58	1
	59	60	2
1	0	5	3
1	0	35	4
1	0	46	5
1	0	3	6
1	0	9	7
1	0	64	8
2	8	10	1
2	9	36	2
2	9	41	3
2	12	66	4
2	13	26	5
2	14	32	6

2	14	62	7	
2	17	11	8	
2	12	97	1	
2	14	73	2	
2	16	21	3	
2	16	36	4	
2	17	86	5	
2	18	35	6	
2	19	49	7	
2	20	21	8	
4	43	49	1	
4	45	17	2	
4	49	10	3	
4	49	71	4	
4	52	12	5	
4	54	34	6	
4	54	46	7	
4	55	60	8	
				1
				1
				1
				1
				1
				1
				1
				1
				3
				3
				3
				3
				3
				3
				3
				3
				7

8
8
8
8
8
8
8
15
15
15
15
15
15
15
15
1
1
1
2
2
2
2
2
1
1
1
1

1
1
1
1
2
2
2
2
2
2
2
2
1
1
1
2
2
2
2
2
2
2
2
2
2
2
2
2
4
4
4
4
4
4
4

4

1
1
1
2
2
2
2
2
4
4
4
4
4
4
4
4
8
8
8
8
8
8
8
8
16
16
16
16
16
16
16
16

1
1
1
1
1
1
1
1
2
2
2
2
2
2
2
2

1
1
1
1
1
1
1
1
2
2
2
2
2
2
2
2

1
1
2
2
2
2
2
2
2
2
2
2
2
2
2
2
2
2
4
4
4
4
4
4
4
4

29	1	3
29	12	4
29	20	5
29	22	6
29	36	7
29	37	8
0	59	1
1	13	2
2	35	3
2	45	4
2	60	5
2	61	6
2	64	7
2	84	8
10	43	1
11	2	2
11	40	3
11	45	4
12	3	5
12	18	6
12	66	7
14	79	8
31	51	1
31	84	2
31	95	3
32	1	4
32	17	5
32	65	6
32	97	7
32	33	8
7	71	1
8	89	2
9	10	3
9	54	4
10	37	5
10	73	6
10	76	7
11	35	8
25	52	1
26	1	2
26	4	3
27	77	4
28	56	5
30	2	6
30	23	7
32	69	8
26	69	1
26	78	2
26	85	3
26	85	4
27	4	5

27	12	6		
27	63	7		
27	68	8		
59	37	1		
59	39	2		
59	64	3		
59	70	4		
59	76	5		
59	82	6		
0	35	7		
0	81	8		
8	25	1		
9	65	2		
11	35	3		
13	23	4		
13	50	5		
14	2	6		
15	12	7		
16	89	8		
13	57	1		
13	96	2		
15	62	3		
16	35	4		
16	53	5		
17	72	6		
17	37	7		
20	38	8		
40	98	1		
43	41	2		
43	53	3		
45	5	4		
46	73	5		
46	78	6		
47	21	7		
49	11	8		
			22	14
			22	19
			22	22
			22	48
			22	53
			22	69
			22	74
			23	0
			48	97
			49	6
			49	47
			49	98
			50	2
			50	17
			50	21
			50	50

1	47	55
1	48	18
1	48	28
1	48	49
1	48	61
1	49	29
1	49	81
1	49	93
3	47	12
3	48	5
3	48	32
3	49	2
3	52	73
3	53	53
3	53	53
3	54	64
7	45	67
7	56	33
7	58	33
7	59	53
8	2	77
8	3	21
8	4	20
8	5	78
14	56	60
15	8	43
15	17	48
15	18	81
15	19	18
15	22	81
15	22	93
15	24	0
	25	44
	25	76
	25	90
	25	93
	26	10
	26	10
	26	11
	26	13
	54	87
	55	24
	55	33
	55	36
	55	37
	55	43
	55	54
	55	66
1	57	92
1	58	21
1	58	42

2	59	10
2	59	36
1	59	80
2	0	80
2	2	86
	27	98
	28	9
	28	18
	28	37
	28	39
	28	46
	28	58
	28	83
1	0	88
1	1	10
1	1	39
1	1	43
1	1	99
1	2	25
1	2	32
1	2	39
2	10	75
2	11	95
2	12	11
2	12	58
2	13	37
2	13	54
2	14	7
2	16	4
	23	96
	23	98
	24	1
	24	9
	24	11
	24	13
	24	19
	24	29
	53	1
	53	17
	53	27
	53	27
	53	56
	53	61
	53	75
	53	80
1	56	42
1	56	82
1	58	57
1	58	80
1	58	99
1	59	58

1	0	20
2	1	80
1	59	44
1	59	50
1	59	74
1	59	84
2	0	31
2	1	59
2	2	9
2	2	12
4	14	97
4	15	89
4	17	67
4	18	5
4	18	36
4	19	64
4	22	43
4	24	52
	25	10
	25	16
	25	38
	25	42
	25	48
	25	65
	25	87
DQ		
	54	47
	54	94
	55	23
	55	31
	55	48
	55	52
	55	99
	56	27
1	58	94
1	59	51
1	59	69
1	59	94
2	0	28
2	1	22
2	2	97
2	2	41
4	7	77
4	10	32
4	11	14
4	11	27
4	12	80
4	15	90
4	17	6
4	19	11
8	30	68

8	36	85
8	38	42
8	40	0
8	43	6
8	45	6
8	47	17
8	49	52
16	23	89
16	33	62
16	35	28
16	35	97
16	44	21
16	46	35
16	49	79
16	52	23
	28	64
	29	71
	29	86
	29	88
	29	3
	29	15
	29	21
	29	47
1	1	5
1	1	18
1	1	27
1	1	33
1	1	56
1	1	85
1	1	87
1	2	2
2	9	74
2	10	68
2	10	79
2	11	62
2	11	75
2	12	23
2	13	0
2	14	7
	29	86
	31	34
	31	38
	31	52
	31	68
	32	20
	32	44
	32	57
1	6	61
1	7	36
1	7	53
1	8	82

1	8	95
1	9	1
1	9	9
1	9	33
2	23	12
2	27	46
2	27	51
2	27	52
2	28	47
2	29	7
2	31	49
2	32	87
	26	32
	26	71
	26	97
	27	4
	27	19
	27	21
	27	26
	27	83
	58	34
	58	73
	59	8
	59	94
1	0	16
1	0	16
1	0	33
1	1	28
2	8	72
2	9	46
2	10	76
2	11	36
2	13	51
2	14	93
2	14	96
2	16	54
2	12	32
2	13	76
2	14	36
2	14	83
2	15	72
2	16	6
2	16	16
2	16	74
4	44	2
4	46	26
4	47	3
4	48	82
4	49	52
4	46	7
4	47	57

	4	47	13

TimeJ2013	PositionJ2013

2
3
4
5
6
7
8
1
2
3
4
5
6
7
8
1
2
3
4
5
6
7
8
1
2
3
4
5
6
7
8
1
2
3
4
5
6
7
8
1
2
3
4
5
6
7
8
1
2
3
4

5
6
7
8
1
2
3
4
5
6
7
8
1
2
3
4
5
6
7
8
1
2
3
4
5
6
7
8
1
2
3
4
5
6
7
8
1
2
3
4
5
6
7
8
1
2
3
4
5
6
7
